# Sequestration of ribosome biogenesis factors in HSV-1 nuclear aggregates revealed by spatially resolved thermal profiling

**DOI:** 10.1126/sciadv.adw6814

**Published:** 2025-06-27

**Authors:** Peter J. Metzger, Tavis J. Reed, Krystal K. Lum, Jordy F. Botello, Lifei Jiang, Clifford P. Brangwynne, Olga G. Troyanskaya, Ileana M. Cristea

**Affiliations:** ^1^ Department of Molecular Biology, Princeton University, 119 Lewis Thomas Laboratory, Princeton, NJ 08544, USA.; ^2^ Lewis-Sigler Institute for Integrative Genomics, Princeton University, Carl Icahn Laboratory, Princeton, NJ 08544, USA.; ^3^ Department of Computer Science, Princeton University, 35 Olden Street, Princeton, NJ 08540, USA.; ^4^ Omenn-Darling Bioengineering Institute, Princeton University, William Street, Princeton, NJ 08540, USA.; ^5^ Department of Chemical and Biological Engineering, Princeton University, 41 Olden Street, Princeton, NJ 08544, USA.; ^6^ Flatiron Institute, Simons Foundation, New York City, NY 10001, USA.

## Abstract

Viruses exploit host cell reliance on compartmentalization to facilitate their replication. Herpes simplex virus type 1 (HSV-1) modulates the subcellular localization of host proteins to suppress immune activation, license viral gene expression, and achieve translational shutoff. To spatially resolve dynamic protein-protein interaction (PPI) networks during infection with an immunostimulatory HSV-1 strain, we integrated nuclear/cytoplasmic fractionation with thermal proximity coaggregation analysis (N/C-TPCA). The resulting expanded depth and spatial resolution of PPIs charted compartment-specific assemblies of protein complexes throughout infection. We find that a broader suite of host chaperones than previously anticipated exhibits nuclear recruitment to form condensates known as virus-induced chaperone-enriched (VICE) domains. Monitoring protein and RNA constituents and ribosome activity, we establish that VICE domains sequester ribosome biogenesis factors from ribosomal RNA, accompanying a cell-wide defect in ribosome supply. These findings highlight infection-driven VICE domains as nodes of translational remodeling and demonstrate the utility of N/C-TPCA to study dynamic biological contexts.

## Teaser

Spatially resolved interaction profiling reveals how HSV-1 disrupts ribosome supply via nuclear chaperone aggregates.

## INTRODUCTION

**Fig. 1.**
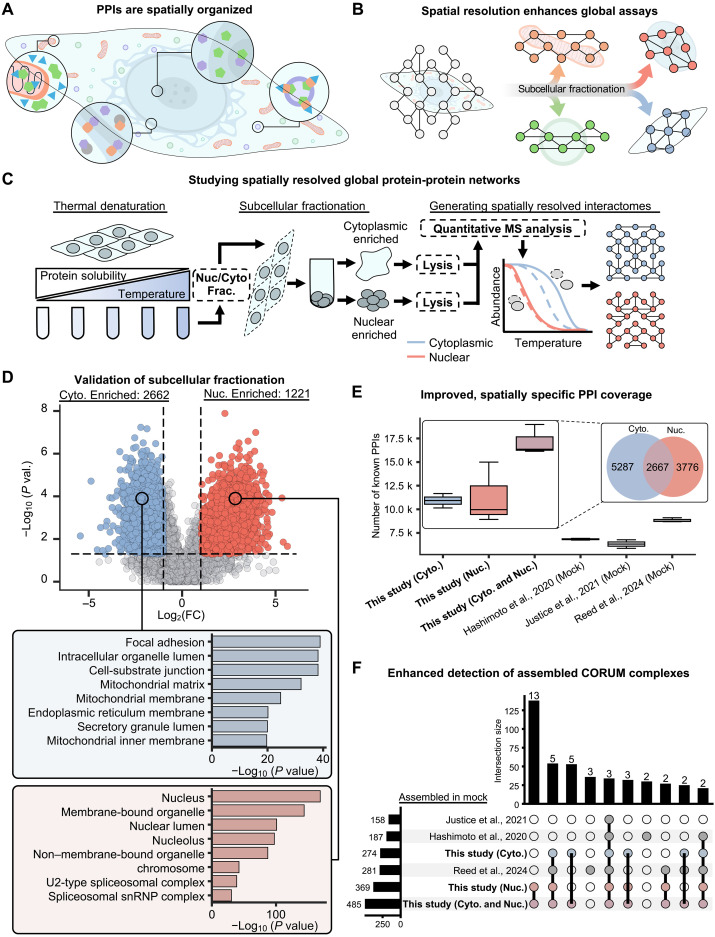
Introducing and evaluating a subcellular fractionation TPCA-based method for studying spatially resolved global PPI networks. (**A**) In cells, PPIs are spatiotemporally regulated. (**B**) Fractionating before performing global assays allows protein interactions to be studied with spatial specificity. (**C**) To couple nuclear-cytoplasmic fractionation with TPCA to probe fraction-specific global PPI networks, the cells are first subjected to thermal denaturation, followed by nuclear-cytoplasmic fractionation, lysis, and MS analysis. Tapioca (*27*) is used to predict PPIs from N/C-TPCA data. (**D**) Volcano plots of N/C-TPCA data represent the relative cytoplasmic and nuclear abundance of the 37°C sample for a given protein. Proteins with enrichment in the cytoplasmic fraction [log_2_(fold change) ≤ −1] are colored blue, and nuclear fraction [log_2_(fold change) ≥1, *P* value ≤0.05] are colored red. Vertical dotted lines represent fold change cutoffs, and horizontal dotted lines represent the *P* value cutoff. Below the volcano plot, the top eight (ranked by *P* value) GO subcellular compartment terms are shown for the cytoplasmic and nuclear fractions. GO term enrichment was performed using Enrichr (*78*). (**E**) The number of previously known PPIs [from BioGrid (*41*), REACTOME (*43*), and Mint (*42*)] that were predicted by Tapioca in the cytoplasmic, nuclear, and combined fractions, as well as in whole-cell samples from the mock conditions of three previous studies (*25*–*27*). In boxplots, boxes show median, 25th, and 75th percentile values, with the line within the box representing the median value, and whiskers represent ±1.5 interquartile range. A Venn diagram compares the most consistently detected known PPIs (detected in two or more replicates). (**F**) UpSet plot comparing CORUM complexes (*39*) assemblies predicted by Tapioca in cytoplasmic, nuclear, and both fractions compared to whole-cell extracts from three previous studies (*25*–*27*). For all experiments depicted, *n* = 3 biological replicates for each temperature.

Cells have evolved vast and interconnected systems of compartmentalization to organize the biochemical reactions that form the basis of their survival and proliferation. The largest compartmental demarcation within most cells is the nuclear envelope, which critically segregates genomic replication and transcription within the nucleus from the milieu of translational, metabolic, and signaling tasks that take place within the cytoplasm. Both the cytoplasm and the nucleus are further organized into an array of membrane-bound organelles and membraneless biomolecular condensates through which such processes are executed (Fig. 1A). As cells respond to stimuli in their environments, resident molecular effectors, including proteins, nucleic acids, lipids, and metabolites, engage in dynamic interactions and exchanges across subcellular compartments. This is especially evident during viral infections, in which viruses and infected host cells compete for control over cellular resources to facilitate infection and activate counteracting defense mechanisms, respectively.

Nearly all viruses coopt every major host cell organelle and induce the formation of specialized viral compartments to promote replication and spread. The nuclear-replicating herpes simplex virus type 1 (HSV-1) is a prototypical example, having coevolved with human hosts over millions of years, thereby acquiring numerous strategies to support lytic replication and establish lifelong latency. HSV-1 infection is accompanied by extensive intracellular remodeling to facilitate virion production, halt host gene expression and protein translation, stimulate viral gene expression, as well as evade immune detection. Examples of intranuclear remodeling mounted by HSV-1 to guide viral genome replication and nucleocapsid egress include compaction and marginalization of host chromatin into the nuclear periphery (*1*, *2*), as well as endoplasmic reticulum (ER) membrane condensation around the nuclear rim (*3*). Disruption of promyelocytic leukemia protein (PML) body nuclear condensates is achieved through the HSV-1 protein ICP0, a viral E3 ubiquitin ligase that marks a variety of nuclear host restriction factors and innate immune signaling proteins for proteasomal degradation during the early stage of infection (*4*). ICP0 dismantles PML body functions in suppressing viral gene expression and replication by targeting constituent proteins for degradation and disrupting the integrity of these membraneless, nuclear condensates (*5*–*9*). During infections with HSV-1 strains lacking ICP0 ubiquitin ligase functions, PML and PML bodies have been shown to contribute to interferon stimulatory activities that restrict the infection (*10*–*12*). Another example is offered by the HSV-1 endoribonuclease, VHS (UL41), which prevents the assembly of stress granules, cytoplasmic accumulations of messenger ribonucleoprotein complexes stalled at translation initiation (*13*, *14*).

These remodeling events are overlaid by viral strategies to recondition the translational status of the cell to suit virus replication and spread. Infectious particles of HSV-1 contain the abovementioned VHS, which induces degradation of both host and viral mRNAs (*15*, *16*). Upon interacting with the translation initiation factor eIF4H, VHS targets and decaps the 5′ ends of mRNAs (*17*). The activity of VHS thus creates a competitive advantage for viral mRNAs as translational substrates and halts antiviral transcriptional responses mounted by host intrinsic immune factors. In an orthogonal strategy to curtail host translation, several HSV-1 proteins relocate polyadenylate-binding proteins from the cytoplasm to the nucleus, where they can no longer support translation via protein-protein interactions (PPIs) with translation initiation factors (*18*–*20*). Together, these examples highlight how compartmental reorganization and shuttling of host factors underlie the intracellular remodeling events necessary for viral replication and inhibition of host defenses.

Integrated knowledge of dynamic protein localization, abundance, and interactions is critical for characterizing the mechanisms underlying intracellular remodeling events during viral infections. Alterations in PPIs can be characterized using various proteomic methods that offer PPI identification in a targeted, i.e., focused on specific proteins of interest, or global manner (*21*, *22*). On the basis of the principle of thermal proteome profiling (*23*, *24*), we and others have leveraged thermal proximity coaggregation (TPCA), coupled with mass spectrometry (MS), to globally characterize virus-host associations in a cell-wide manner throughout virus replication cycles (*25*–*28*). This approach is based on the principle that proteins that interact with one another behave as a unit, thereby co-aggregating upon heat denaturation and displaying highly correlated thermal solubility profiles. This biochemical property of protein complexes enables TPCA to globally monitor PPIs for endogenous proteins in a given cell or tissue sample (*23*, *24*, *29*). To date, TPCA has only been applied to whole-cell samples, presenting challenges for capturing low-stoichiometry complexes or localization-dependent PPIs. A modified TPCA approach that quantitatively monitors PPIs, while also simultaneously capturing localization and shuttling dynamics, would therefore offer insights into dynamic intracellular remodeling with spatial and temporal specificity (Fig. 1B).

Here, we integrate a subcellular spatial dimension with TPCA and use this method to functionally characterize a virus-induced biomolecular condensate in the nucleus. Integration of nuclear-cytoplasmic fractionation within a thermal denaturation workflow (N/C-TPCA) offered the greatest coverage of PPIs reported for TPCA studies thus far, resolved specificity for interactions obscured in conventional whole-cell extract analyses, and provided compartment-specific information for complex assemblies. Specifically, we interrogate nuclear-cytoplasmic shuttling events and intranuclear movement of host and viral proteins throughout infection of primary human fibroblasts with a mutant strain of HSV-1 that is known to potentiate an intrinsic immune signaling response orders of magnitude higher than wild-type (WT) HSV-1 (*30*, *31*). Investigation of compartmental abundance trends, in conjunction with multipartite protein interaction networks, revealed the accumulation of dozens of host chaperones within the nucleus, reflecting the formation of virus-induced chaperone-enriched (VICE) domains (*32*, *33*). Designing an extended interaction network approach to analyze TPCA-predicted PPIs led us to experimentally confirm that HSV-1–driven depletion of ribosomal subunits and polysomes is accompanied by VICE domain recruitment of host factors involved in multiple major steps of ribosome biogenesis. We further show that VICE domains spatially decouple these factors from premature ribosomal RNAs (rRNAs), which they colocalize with in the nucleolus under homeostatic conditions to support ribosome biogenesis (*34*). Using pulse chase imaging to trace the origin of ribosomal structural elements we observed within VICE domains, we show that distinct populations of both nascent nuclear and mature cytoplasmic ribosomes are recruited to these HSV-1–driven nuclear aggregates. Overall, our results demonstrate the utility of N/C-TPCA to capture suborganellar remodeling events and provide a perspective on the function of VICE domains as node in the translational remodeling of infected cells.

## RESULTS

### Generating nuclear/cytoplasmic resolved PPI networks using TPCA

To capture the dynamic nuclear/cytoplasmic coordination of PPI networks, we integrated nuclear/cytoplasmic fractionation within a TPCA experimental workflow. To facilitate this, we had to consider that performing a lysis step before thermal denaturation has previously been shown to alter the measured protein melting curves and predicted PPI profiles (*29*). Thus, we opted to perform thermal denaturing first to better maintain PPIs before subcellular fractionation (Fig. 1C). Cell lysis in 0.2% NP-40 facilitates the dissolution of the plasma membranes while retaining the nuclei intact (*35*). Monitoring localization markers following thermal denaturation and nuclear/cytoplasmic fractionation shows that these markers retain their separation and enrichment in their corresponding subcellular compartment across denaturation temperatures and HSV-1 infection time points [3, 8, and 15 hours post infection (hpi)] (fig. S1A).

Following the separation of the cytoplasmic and nuclear fractions by centrifugation, each fraction was processed through a TPCA MS workflow. Recent reports from several groups, including ours, have identified a range of denaturation temperatures that provide protein melting profiles that can be used for downstream PPI prediction. Within this optimal temperature range, we and others have shown that it is feasible to reduce the number temperature points to five while retaining PPI prediction quality (*27*, *36*). Here, this five-temperature TPCA workflow enabled us to increase sample multiplexing and, thus, the analysis throughput, making the analysis of the nuclear and cytoplasmic fractions experimentally tractable. Using the 37°C fractions as proxy for nuclear and cytosolic proteomes, we confirmed by MS the corresponding enrichment of proteins with known or unknown nuclear localization, respectively (Fig. 1D). This observation supported our Western blot analysis, indicating that performing thermal denaturation before subcellular fractionation does not disrupt the efficacy of the fractionation. To further validate this observation, we performed subcellular fractionation without prior thermal denaturation, in the same experimental context, and analyzed these samples using label-free data-independent acquisition (DIA) MS. We observed appreciable correlation in the relative abundances of proteins in the nuclear and cytoplasmic fractions between samples that have been fractionated with (i.e., TPCA MS) or without (i.e., DIA MS) prior thermal denaturation (fig. S1B). Given the presence of populations of proteins that also showed poor correlation, we next sought to better understand the subsets of proteins that may be most affected by fractionation or thermal denaturation within the TPCA workflow. Using the human protein atlas to assign localizations to proteins, we analyzed the correlation between thermally denatured and non-thermally denatured proteins per localization (fig. S1C) (*37*). We observed good correlations for proteins localized to the nuclear membrane, nucleoli, and nucleoplasm, and lower correlations for mitochondrial and ER-localized proteins. This suggests that the TPCA workflow may induce some localization-specific alterations to observed protein abundances, which is a noteworthy consideration when applying this N/C-TPCA workflow.

The thermal stability of proteins is determined by a number of factors, including the biophysical characteristics and interactions with proteins, lipids, and nucleic acids (*24*, *38*). Therefore, changes in thermal stability can represent a range of alterations in the behavior or functionality of a protein. When comparing the thermostabilities of proteins detected in both nuclear and cytoplasmic fractions, we found localization-specific thermostability profiles (fig. S1D). Proteins enriched in the nuclear fraction were more thermally stable in the cytoplasmic fraction, and vice versa. Thus, it would appear that these proteins in particular are engaging in distinct interactomes between fractions. This highlights the potential for subcellular fractionation to enable TPCA to reveal more holistic PPI interactomes.

Given the observation of distinct thermal stability between fractions, we next investigated differences in the predicted PPI interactomes. We used Tapioca, a machine learning–based framework that integrates TPCA data with tissue-specific functional networks and protein properties to predict context-specific PPIs (*27*). A select subset of CORUM complexes (*39*) were used as gold standard (*40*) to inspect the quality of PPIs predicted from each fraction. A synthetic whole cell was generated by summing the raw MS data for proteins in each fraction. We observed overall similar PPI prediction quality, with the cytoplasmic fraction showing a slightly lower median area under the precision-versus-recall curve (AUPRC) but lower overall variability in AUPRC between replicates (fig. S1E). Next, when assessing known PPIs from BIOGRID (*41*), MINT (*42*), and REACTOME (*43*) databases, we found that more known PPIs were predicted in both the nuclear and cytoplasmic fractions than in previous TPCA studies (*25*–*27*) from our laboratory that used whole-cell workflows (Fig. 1E). Furthermore, when directly comparing the nuclear and cytoplasmic fractions, we observed that most known PPIs were uniquely predicted by each fraction. These observations highlight the usefulness of fractionation to improve the depth of the PPI network coverage, in conjunction with spatial information.

### Protein complexes demonstrate distinct assembly and behavior between subcellular locations

To accomplish a diverse array of biological functions, proteins form multimembered complexes with other proteins. The CORUM complex database (*39*) is a repository of thousands of such complexes that have been defined experimentally. As the composition of CORUM protein complexes can be spatially regulated in response to stimuli, we determined their representation in our nuclear and cytoplasmic fractions and compared our results to prior reports (Fig. 1F). First, we filtered our predicted PPIs datasets, retaining complexes that have at least 50% of their protein subunits detected. Calculating average Tapioca scores (see Materials and Methods) for all subunit pairs within a complex, we determined which CORUM complexes are predicted to be assembled in each fraction. To allow further assessment of our results, we repeated this CORUM complex scoring method using TPCA data from previous studies (Fig. 1F). We found that, together, nearly twice as many CORUM complexes were identified as assembled in the nuclear and cytoplasmic fractions than in our previous studies using whole-cell TPCA workflows. When assessing localization-dependent complex detection, we observed that fractionation allowed the identification of distinct subsets of assembled complexes between the nuclear and cytoplasmic fractions.

**Fig. 2.**
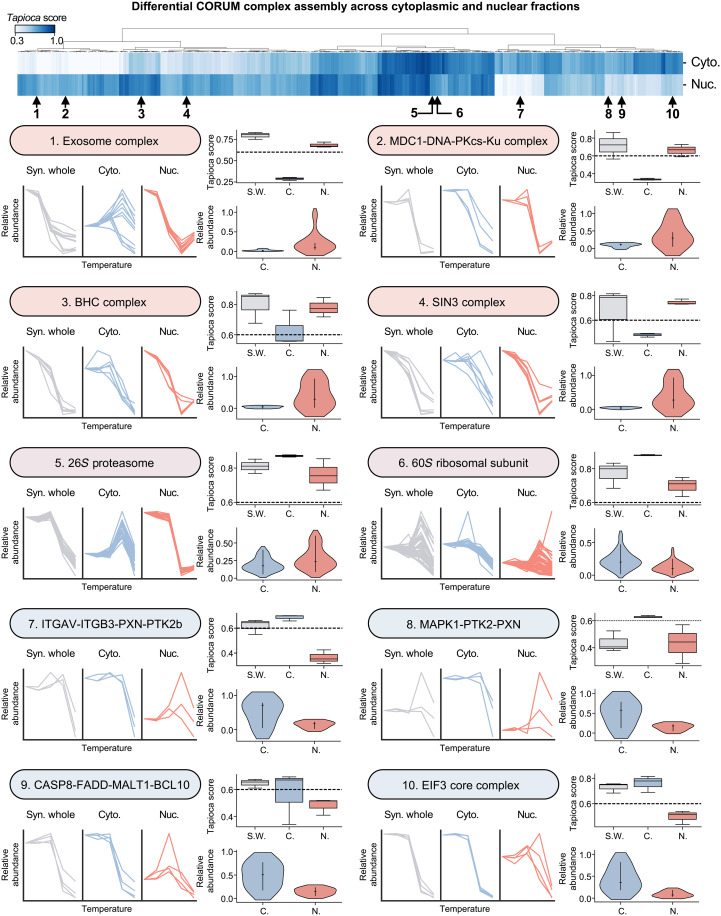
N/C-TPCA enables detection of spatially specific CORUM complex assembly and melting curve behavior. A heatmap of the Tapioca scores of CORUM complexes (*39*) in the cytoplasmic and nuclear fractions is shown. The table of CORUM complexes and their scores can be found in the Supplementary data. Examples of melting curve behavior, Tapioca scores, and relative abundance were generated for 10 of these complexes. For these vignettes, a synthetic whole cell (Syn. Whole, S.W.) fraction was created by summing the reference normalized cytoplasmic (Cyto., C.) and nuclear (Nuc., N.) melting curves before further normalization. In the melting curve plots, each line represents the replicate-averaged melting profile for a single protein within the CORUM complex. CORUM complexes were predicted to be assembled if they achieved an average score greater than or equal to 0.6. This cutoff is shown as a horizontal dotted line in the boxplots. In boxplots, boxes show median, 25th, and 75th percentile values, with the line within the box representing the median value, and whiskers represent ±1.5 interquartile range. The reference normalized value at the 37°C for each protein within the complex was used to generate the relative abundance violin plots. For violin plots, the white dot represents the median, the thick black bar represents the ±1.5 interquartile range, and the thin gray line represents the total range, excluding outliers. For all experiments depicted, *n* = 3 biological replicates for each temperature.

We next more closely inspected the differences in CORUM complex assembly and behavior between the nuclear and cytoplasmic fractions, as well as in the generated synthetic whole-cell fraction (Fig. 2). We clustered complexes by their predicted localization-enriched assembly. Selecting representative protein complexes from these clusters, we analyzed differences in thermal stability curves, average Tapioca scores, and the relative abundances of subunits between cytoplasmic and nuclear fractions. Some complexes with subunits predominantly localized to one fraction, as determined by nuclear/cytoplasmic proteomes assessment at 37°C, also displayed localization-specific assembly, as the TPCA-derived curve data and Tapioca scores predicted their assembly only in that fraction or in synthetic whole cell. Examples include the exosome (DIS3L, EXOSC1, EXOSC2, EXOSC3, EXOSC4, EXOSC5, EXOSC6, EXOSC7, EXOSC8, EXOSC9, EXOSC10, and MPHOSPH6), involved in the degradation of RNA, and EIF3 (EIF3A, EIF3B, EIF3G, and EIF3I), involved in translation initiation, complexes that displayed exclusively nuclear and cytoplasmic assemblies, respectively, in keeping with their respective functions in histone deacetylation and translation initiation. For other complexes, we observed that while the entire complex was only assembled in one fraction, the assembly of distinct subcomplexes could be seen in the other fraction. This is exemplified by the DNA repair–related MGC1-DNA-PKcs-KU complex, whose subunits showed complete assembly in the nuclear fraction, while appearing to form two distinct subcomplexes in the cytoplasmic fraction. In this case, the synthetic whole fraction predicted the entire complex to be assembled; however, some complexes required nuclear/cytoplasmic fractionation to detect their assembly. Such was the case for the MAPK1-PTK2-PXN complex, involved in focal adhesion–related signaling, which was only detected as assembled within the cytoplasmic fraction. In some cases, such as for the LAP1-TOR1A and FGFR2-c-Cbl-Lyn-Fyn complexes, the fractionation enabled the detection of complex assembly in the subcellular compartments where they exhibited lower abundances (fig. S2). Even for complexes identified to be assembled in all fractions, such as the 26S proteasome, distinct thermal stability curves were observed, suggesting that these complexes participated in different interactomes in these distinct subcellular locations. Overall, our results show that integration of subcellular fractionation with TPCA results in an increased resolution of CORUM complex detection and localization elucidation, boosting fraction-specific signals, while dampening the cross-talk interference from the other compartment.

### Spatially resolving the dynamic behavior of protein interactomes throughout HSV-1 infection

**Fig. 3.**
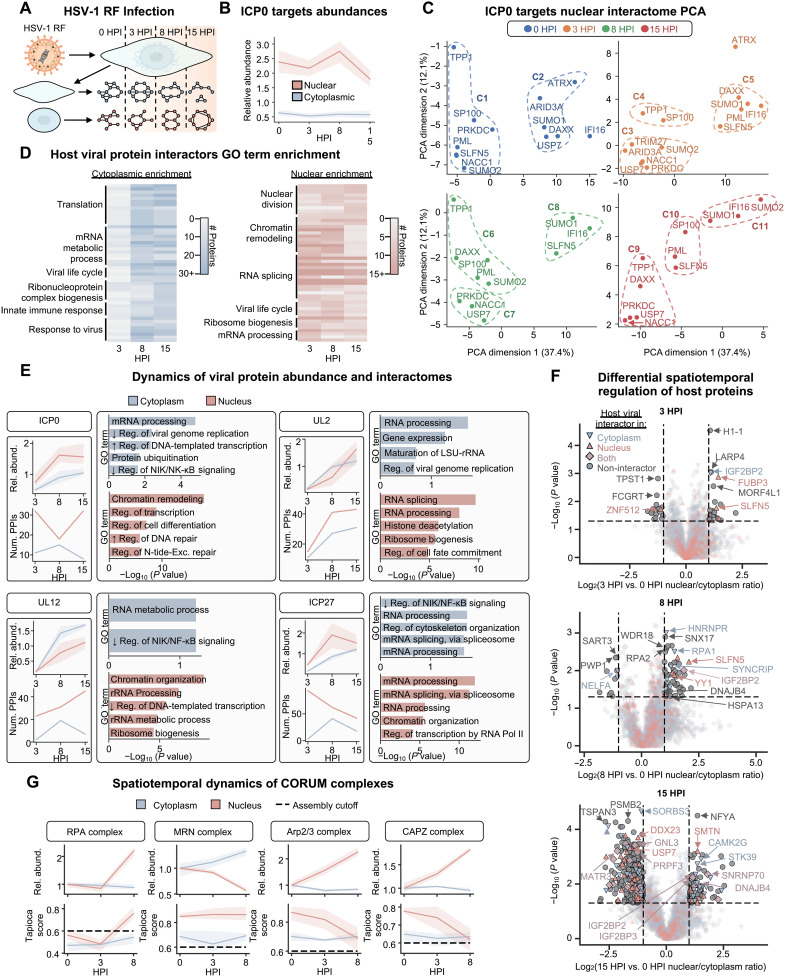
Leveraging N/C-TPCA to characterize the spatiotemporal regulation of global PPI networks during immunostimulatory HSV-1 infection. (**A**) Schematic representation of HSV-1 RF infection N/C-TPCA experiment. (**B**) Nuclear and cytoplasmic relative abundance of ICP0 host targets throughout the infection time course. (**C**) PCA on ICP0 host target interactome across all time points. Interactors were included if they achieved a Tapioca score of at least 0.3 with at least one target. K-means clustering (K = 3, for 0, 8, and 15 hpi, or K = 4 for 3 hpi) was used to cluster the ICP0 target proteins. The first and second PCA dimensions and clusters (dotted outline) are shown. (**D**) Temporal GO term enrichment [using HumanBase (*77*)] of host interactors of viral proteins in the nuclear and cytoplasmic fractions. (**E**) Vignettes of viral protein relative abundance, number of PPIs, and GO term enrichment of all host interactors. In PPIs plot, the line represents the number of PPIs with a minimum median Tapioca score of 0.7. Top 5 GO terms [using Enrichr (*78*)] were ranked by *P* value, are shown. (**F**) Protein nuclear/cytoplasmic ratio at different time points of infection (3, 8, and 15 hpi) compared to mock (0 hpi). Each marker represents the average abundance across three replicates for a single protein and is colored by the fraction in which it is predicted to interact with a viral protein. Cutoffs, *P* value (0.05) and fold change, (±1) are shown as dashed lines. (**G**) Temporal dynamics of CORUM complex (*39*) abundances and Tapioca scores. Complexes are considered assembled if they pass the cutoff score (0.6). For all line plots, the solid line represents the median value and the shaded region is the 95% confidence interval. For all experiments depicted, *n* = 3 biological replicates for each temperature and infection time point.

Having demonstrated the feasibility and benefits of coupling nuclear/cytoplasmic fractionation with TPCA for localization-specific global interaction profiling, we next leveraged this technique to study a viral infection, which represents a biological context that is dynamic in both the protein interaction state and subcellular organization of the proteome. We performed a spatiotemporal interactome study across the replication cycle of the prominent human pathogen, HSV-1 (Fig. 3A). To capture dynamic processes at the interface of host-pathogen interactions, we used an HSV-1 strain that induces the activation of host immune signaling while still progressing through the entire replication cycle. This allowed us to study the repertoire of protein interactomes and dynamic localization behavior underlying both virus replication processes and host defense responses. Specifically, we chose to use the ICP0-RF strain of HSV-1, which contains inactivating point mutations in the E3 ubiquitin ligase domain of the immediate-early viral protein ICP0, rather than an ICP0-null strain, to only minimally impair the progression of infection (*30*, *44*). This E3 ligase domain normally functions to mark a suite of antiviral host proteins in the nucleus for ubiquitin-mediated degradation (*4*). Thus, infection with ICP0-RF permits the investigation not only of the behavior of these degradation targets but also of the downstream immune signaling response.

To perform spatially resolved thermal profiling across the HSV-1 replication cycle, we synchronously infected fibroblasts, as previously described (*45*), to minimize heterogeneity derived from an asynchronous viral entry. We opted to use a relatively high multiplicity of infection (MOI) of 5 for this experiment, given observations of reduced permissiveness and focus-forming ability of ICP0-deficient HSV-1 strains in human foreskin fibroblasts (HFFs) (*46*), and to align with prior reports using these strains (*47*, *48*).

We first analyzed the number of predicted PPIs, both known and de novo, that were detected at time points representing distinct stages of the HSV-1 replication cycle (fig. S3, A to D). An overall decrease in total PPIs and increase in virus-virus and virus-host PPIs was observed, which is expected given the HSV-1–induced degradation of host mRNAs and proteins during infection (*4*, *16*, *49*). Despite the lower total number of PPIs as infection progressed, we found that the interactomes of both viral and host proteins remain highly compartment-specific throughout infection (fig. S3E).

We next turned our focus to host proteins targeted for degradation by the viral protein ICP0. As expected, these factors displayed primarily nuclear localization and were spared from degradation (Fig. 3B), with several proteins also exhibiting increased nuclear abundance in infected cells (e.g., IFI16 and SLFN5; fig. S3F). To explore the temporal relationship between the interactomes of these proteins, we performed principal components analysis (PCA) throughout the infection (Fig. 3C and fig. S4A and S5). For this PCA, each observation was a specific ICP0 degradation target at a specific hpi. Variables were the Tapioca scores of interactions between the given ICP0 degradation target other proteins in the dataset that met a minimum score threshold in at least one time point. Thus, broadly, it is expected that points close in PCA space have similar interactomes, while points far apart have different interactomes. While most of the produced PCA dimensions seemed to be largely dependent on interactomes, we note that the third PCA dimension seems to separate by hpi. For each time point, clustering of the resulting PCA values (using K-means clustering using the PCA values with K = 3 for 0, 8, and 15 hpi and K = 4 for 3 hpi) and analysis of the common interactors of ICP0 target proteins within given clusters allowed assessment of the temporal movement of these proteins between clusters (fig. S4B). This analysis revealed several dynamic features of the relationship between ICP0 degradation targets, particularly IFI16, PML, and SLFN5. In uninfected cells, the failure of IFI16 to cluster with other targets likely reflects its homeostatic localization to the nucleolus, in contrast to the nucleoplasmic localization of most other factors included in this analysis (*10*, *37*, *47*). However, this relationship changes as early as 3 hpi, a time point at which IFI16, PML, and SLFN5 would normally begin to experience proteolytic degradation following their inclusion in nuclear puncta containing ICP0 (*47*). By 3 hpi, SLFN5, PML, and DAXX exhibit a shift in PCA space to cluster with IFI16. Then, as viral replication and innate immune signaling proceed during infection with ICP0 RF, by 8 hpi, the interaction profile of SLFN5 remains correlated with IFI16, while those of PML, DAXX, and SP100 diverge to cluster with one another. Following this divergence, we identify shared interaction partners between IFI16 and SLFN5 at both 3 and 8 hpi, including host immune factors (PQBP1, NFKB1, NBN, TANK, and OASL) and viral factors that antagonize innate immune signaling (VP22 and UL46) (*50*, *51*). Shared interactions also included proteins active at the nuclear periphery, such as host tethering factors (SUN2 and TOR1AIP1) and viral capsid assembly factors (NEC1 and CVC1), potentially implicating IFI16 and SLFN5 in suppressing viral encapsidation or nuclear egress.

Next, to understand the biological processes being directly targeted by viral proteins through virus-host interactions more broadly, we performed GO term enrichment on the host interactors of viral proteins in each fraction at each infection time point (Fig. 3D and figs. S6 and S7). We observed RNA-related processes, such as “RNA splicing” and “mRNA metabolic process,” as commonly targeted in both fractions across most time points. Also prominent in both fractions were translation-related terms, which included “ribosomal large subunit biogenesis” in the cytoplasm and “ribosome biogenesis” within the nucleus. Despite the targeting of these common biological processes, our previous observation of highly fraction-specific viral interactomes suggests that distinct components of these biological processes are being targeted across subcellular locations. Unique to the cytoplasmic fraction, we observed temporally regulated viral protein interactions with host proteins involved in innate immunity, mitochondrial gene expression, and lipid response. Within the nucleus, we observed temporal enrichment of nuclear division, chromosome regulation, and organelle remodeling, in agreement with the known viral-induced reorganization of the host nucleus observed during HSV-1 infections (*1*, *3*, *52*, *53*). At 15 hpi, we observed the appearance of terms like “plasma membrane raft assembly” and “membrane to membrane docking,” which may be associated with the degradation of the nuclear membrane and release of assembled capsids associated with this time point (*52*, *53*).

Delving deeper in the identified spatiotemporal virus-host interactome, we noted the fraction-dependent alteration in interactions for viral proteins known to be critical in the HSV-1 replication cycle (Fig. 3E). As expected, the relative abundance of the viral proteins steadily increased in both fractions throughout the infection, albeit with different rates and temporal profiles. By generating profiles of temporal and spatial viral protein abundances and PPIs, we observe that changes in abundance and PPI numbers are not coupled, highlighting distinct mechanism of viral protein regulation. Given our use of an immunostimulatory virus strain, we examined the functional classes represented within PPIs of viral proteins with known roles in immune evasion. In agreement with their functions (*54*, *55*), the viral proteins ICP0 and ICP27 displayed enriched interactions with host proteins involved in “negative regulation of NIK/NK-kappa B signaling” in the cytoplasm. Furthermore, the cytoplasmic interactome of ICP0 was enriched for “protein ubiquitination.” Within nuclear fractions, the ICP0 interactome was enriched for terms relating to chromatin remodeling, transcription, and DNA repair. This likely reflects the ability of ICP0 to target immune factors, such as IFI16, which bind to viral DNA as part of the host immune response to HSV-1 infection (*56*). An unexpected observation was the enrichment of the nuclear interactome of alkaline nuclease (AN) for “rRNA processing” and of both UL12 and UL2 for “ribosome biogenesis,” suggesting that these proteins might play roles in translation, in addition to their known roles in viral genome replication as a viral endo- and exonuclease protein (UL12) and excision of uracil residues from DNA (UL2).

Having analyzed interaction networks and pathways targeted by viral proteins, we next overlaid measurements of spatiotemporal alterations in host protein abundances to uncover dynamic regulation and possible translocation events (Fig. 3F). As the infection progressed, we observed an increase in the number of proteins that demonstrated significant [|log_2_(fold change)| ≥ 2, *P* value ≤0.05] changes in nuclear-to-cytoplasmic abundance ratios, becoming more enriched in either the cytoplasmic or the nuclear fractions compared to uninfected cells. Many of these host proteins were also predicted in our dataset to form interactions with viral proteins in either the cytoplasmic, nuclear, or both fractions, suggesting that viral factors may promote some of these dynamic events. Consistent with previous observations, functional cohorts that became more cytoplasmic during infection included chromatin remodeling factors and splicing factors, while cohorts exhibiting a relative increase in nuclear abundance contained members of the actin cytoskeletal machinery, PABPs, and DNA replication and repair factors.

To further understand the dynamic behavior of CORUM complexes, we compared the temporal regulation of subcellular enrichment and complex assembly (i.e., Tapioca scores). Similar to viral proteins, this comparison indicated that abundance and interaction trends are not always predictive of one another (Fig. 3G). For example, we observed a simultaneous increase in both the nuclear abundance level and complex assembly score for the heterotrimeric replication protein A (RPA) complex by 8 hpi, in line with previous observations that this complex supports viral DNA replication by functioning in ATR-dependent DNA damage repair (DDR) (*57*). In contrast, another DDR-related DNA-binding complex, Mre11-RAD50-NBN (MRN), appeared to remain stably assembled in both nuclear and the cytoplasmic fractions throughout infection, while its relative nuclear and cytoplasmic abundances displayed opposite decreasing and increasing trends, respectively. This suggests that HSV-1 infection may alter the function of the MRN complex by inducing its relocalization to the cytoplasm instead of triggering complex disassembly. Our data also reflect known HSV-1–driven accumulation of actin cytoskeleton elements in the nucleus (*58*). Despite the robust increase in nuclear abundance observed for members of the Arp2/3 and CAPZ complexes, important for actin regulation, these complexes show a steady decrease in their predicted nuclear assembly, whereas their predicted formation in the cytoplasm remains consistent. Together, these results highlight the capability of N/C-TPCA to offer insight into the mode of regulation of protein complexes in dynamic contexts by simultaneously reporting compartmental abundance status and compartment assembly status.

### HSV-1 drives the accumulation of host chaperones in the nucleus

**Fig. 4.**
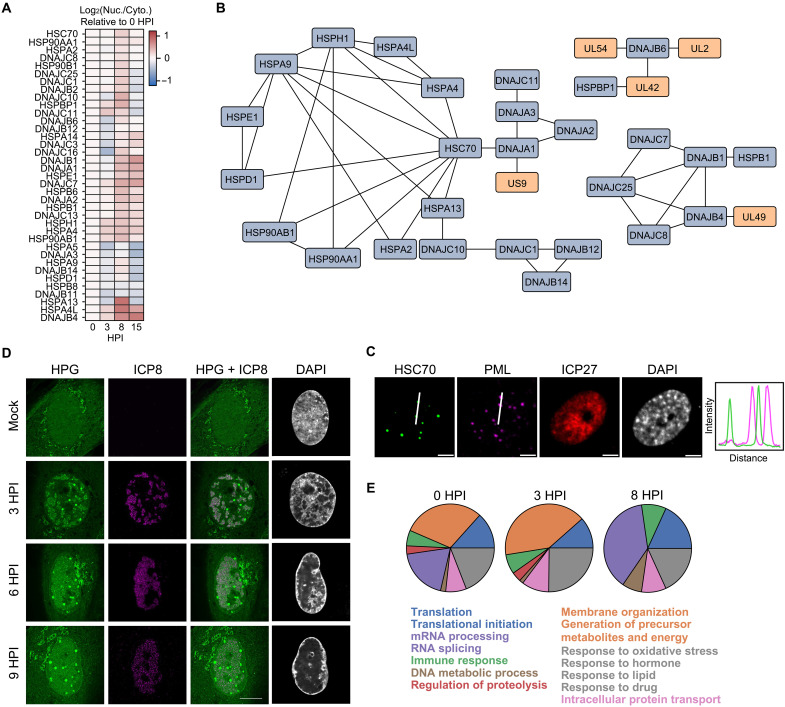
Chaperone proteins exhibit dynamic recruitment to the nucleus and engage in specific nuclear interaction networks during HSV-1 infection. (**A**) The temporal dynamics of chaperone protein relative abundance ratios (nuclear/cytoplasmic) relative to their ratio at 0 hpi. (**B**) Network of interacting chaperone and viral proteins. Proteins are connected if they are predicted to interact with one another at any time point (0 to 15 hpi). Proteins are colored as belonging to host (blue) or virus (orange). (**C**) HFFs were infected with ICP0-RF HSV-1 (MOI 5) and collected at 8 hpi, followed by immunofluorescent staining against HSC70, PML, and ICP27. Scale bar, 5 μm. (**D**) HFFs were mock infected or infected with WT HSV-1 (MOI 5) and collected at 3, 6, or 9 hpi. One hour before collection, the cells were depleted of l-methionine for 30 min followed by incubation with L-HPG for 30 min to label nascent proteins, after which sequential click-based fluorescent conjugation and immunofluorescent staining were performed. (**E**) Pie charts showing the number of proteins associated with a given GO term at different time points. GO term enrichment was performed using HumanBase (*77*) on the set of host proteins predicted to interact in the nucleus with at least three chaperone proteins present in the network shown in Fig. 4B. These GO terms were then ranked by the maximum number of proteins associated with a given term across all time points of infection. The top 50 GO terms were manually clustered, and the total number of proteins belonging to each cluster was used to generate pie charts.

During our analysis of proteins that displayed dynamic behavior during HSV-1 infection, we identified subsets of host proteins for which their altered abundances pointed to likely movements between the nucleus and the cytoplasm. Prominent among these were chaperone proteins, including proteins in the HSP40, HSP70, and HSP90 families. By analyzing their temporal nuclear/cytoplasmic abundance ratios, we found that the phenotype of increased nuclear abundance peaked for most of these factors at 8 hpi (Fig. 4A). To understand whether their correlation in spatial abundance is also reflected in the interaction space, we monitored the temporal and spatial connectivity between these factors. At 8 hpi, an interaction network consisting of host chaperone proteins and several viral proteins that increased in relative nuclear localization became evident (Fig. 4B). We were intrigued to see that the protein that displayed the largest number of connections within this network was HSC70 (also named HSPA8), a protein with both anti- and proviral roles during a range of viral infections (*59*).

HSC70 is known to be a marker for the appearance of VICE domains, punctate nuclear domains that arise specifically during infection with HSV-1 (*32*, *33*, *60*, *61*). Hence, we predicted that the temporality of this chaperone enrichment in the nucleus coincides with VICE domain formation. However, to our knowledge, the formation of VICE domains has not been examined in ICP0-deficient infection contexts. Therefore, using microscopy, we observed the formation of nuclear HSC70 puncta that were distinct from PML puncta at 8 hpi following infection with ICP0-RF HSV-1 (Fig. 4C). This is in agreement with the knowledge that, at this time point of HSV-1 infection, HSC70 and PML form puncta that are similar in appearance but spatially distinct (*62*). VICE domains have also been observed to intensely concentrate nascent proteins (*62*), a finding that we recapitulated over multiple time points of HSV-1 infection using l-homopropargylglycine (HPG) to specifically label newly synthesized proteins. In agreement with prior findings, signal corresponding to nascent proteins formed intense puncta matching the appearance of VICE domains and studding the perimeter of ICP8 replication compartments (Fig. 4D).

VICE domains are understood to function as sites of nuclear protein quality control that capture model misfolded proteins and which sometimes include ubiquitinated substrates and proteasomal components. However, there is still limited knowledge about the molecular components or functional consequences of VICE domains and which, if any, other host proteins are localized to these structures. Therefore, we sought to use the temporal, coordinated localization captured by our interaction profiling of HSV-1–infected nuclei to further elucidate the composition of VICE domains. We performed temporal GO term enrichment on host proteins that interacted in the nucleus with at least three of the chaperone proteins identified in Fig. 4B, observing clear temporality in enrichment (Fig. 4E). In uninfected cells, chaperone protein interactions consisted of a wide range of biological processes, including splicing, gene expression, translation, immune regulation, ER and mitochondrial processes, and protein transport. However, by 8 hpi, this enrichment greatly narrowed, encompassing terms relating to translational initiation and splicing. Furthermore, the loss of interactions with the ER and mitochondrial processes by 8 hpi further reflects the relocalization of these chaperones from the cytoplasm to the nucleus to engage different functional networks of interaction partners. When considering interactions with translation-related proteins that were uniquely gained upon infection, we uncovered large and small ribosomal subunit proteins, as well as factors involved in translation initiation and regulation of rRNA processing (fig. S8). Several proteins involved in mRNA processing and stabilization were also observed. Together, these findings indicate that a broader suite of host chaperones may be recruited to VICE domains upon HSV-1 infection than previously appreciated. Moreover, this analysis predicts that the shared interactome of nuclear-enriched chaperones narrows as infection progresses, potentially reflecting the coalescence of host factors with roles in mRNA regulation and translation within VICE domains.

### VICE domains segregate molecular effectors of ribosome biogenesis

**Fig. 5.**
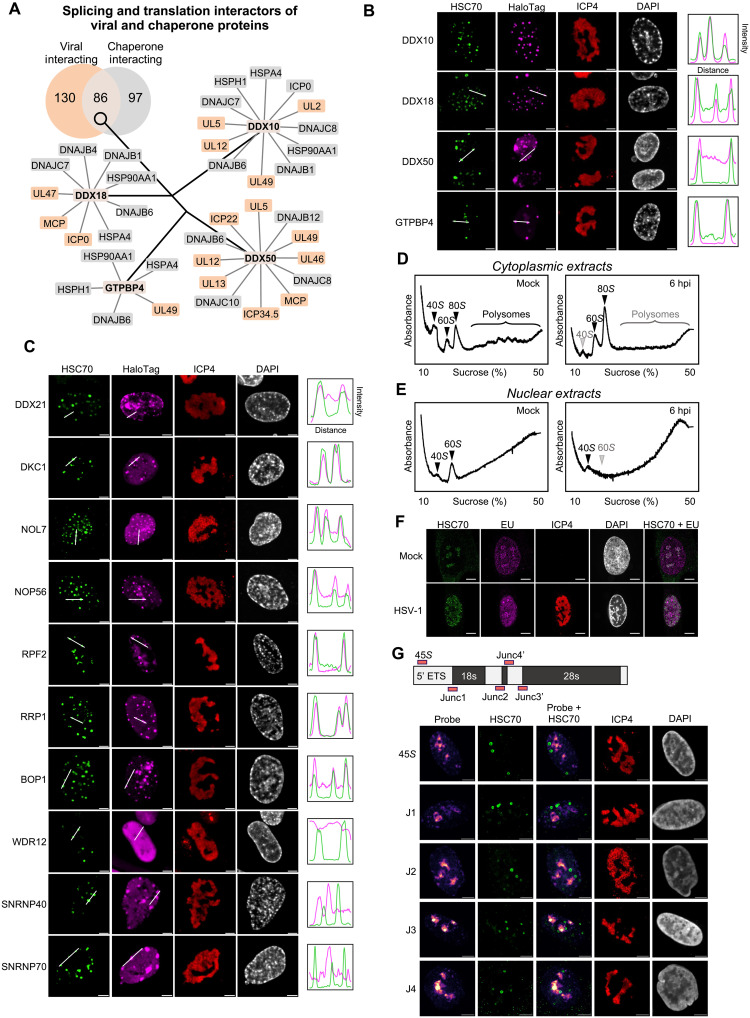
VICE domains enrich nucleolar ribosome biogenesis factors while excluding nascent RNAs. (**A**) Networks in which the center protein is a host protein that interacts connected to its specific chaperone and viral interactors. Each network is shown as a separate entity, and chaperone-chaperone, chaperone-virus, and virus-virus edged are excluded for simplicity. (**B** and **C**) HFFs stably expressing the indicated HaloTag fusion protein were mock infected or infected with WT HSV-1 (MOI 5) and collected at 8 hpi, followed by immunofluorescent staining against HSC70 and ICP4. Scale bar, 5 μm. (**D** and **E**) HFFs were mock infected (left) or infected with WT HSV-1 (MOI 5) (right). At 6 hpi, cytoplasmic (D) and nuclear (E) extracts were subjected to sucrose gradient fractionation. (**F**) HFF cells were mock infected or infected with WT HSV-1 and collected at 6 hpi. Thirty minutes before collection, 5-ethynyl uridine was added to the media to label nascent RNA, after which sequential click-based fluorescent conjugation and immunofluorescent staining was performed against HSC70 and ICP4. Scale bar, 10 μm. (**G**) RNA FISH probes were designed to span splicing junctions absent in mature transcripts. HFFs were mock infected or infected with WT HSV-1 (MOI 5) and collected at 8 hpi, after which sequential fluorescent in situ hybridization and standard immunofluorescence were performed against the indicated probe and HSC70. Scale bar, 5 μm.

Located at the edge of HSV-1 replication compartments, VICE domains occupy a site of dynamic molecular exchange at which viral proteins dictate a milieu of DNA replication, transcription, and posttranslational processing. To narrow the focus of our investigation, we therefore examined predicted VICE-associated chaperone interactors related to splicing and translation that share interactions with multiple viral proteins. At 8 hpi, more than 80 proteins linked to splicing and translation-related biological processes were found at the intersection of associations with chaperone and viral proteins, suggesting a broader modulation of these processes in the nucleus (Fig. 5A). Several host proteins with roles in ribosome biogenesis stood out. DDX10, DDX18, and DDX50 are splicing factors known to regulate ribosomal DNA accessibility as well as rRNA transcription and cleavage (*63*–*65*), while GTPBP4 is an integral component of large ribosomal subunit biogenesis (*66*). Using microscopy, we confirmed the prediction of our chaperone interaction analysis, observing colocalization of DDX10, DDX18, DDX50, and GTPBP4 with VICE domains upon infection with HSV-1 (Fig. 5B).

The temporal enrichment in translation and splicing factors, in conjunction with ribosomal structural proteins, led us to ask whether chaperone proteins, specifically within VICE domains, may influence ribosome biogenesis. Hence, we used microscopy to analyze additional factors involved in rRNA processing (DDX21, DKC1, RRP1, BOP1, and WDR12), large (NOP56 and RPF2) or small (NOP56 and NOL7) subunit assembly, and spliceosome components (SNRNP40 and SNRNP70). WDR12 and BOP1, together with PES1, form the canonical PeBoW complex, a critical factor involved in large ribosomal subunit biogenesis. Our results suggest the recruitment of BOP1 and PES1, but not WDR12, to VICE domains (Fig. 5C and fig. S8), which correlates with the steady decline in assembly status of the PeBoW complex in both the nucleus and cytoplasm as infection progresses (data file S1). Notably, of the proteins included in this imaging study, only those that displayed nucleolar localization in uninfected cells (fig. S9) colocalized with HSC70 and VICE domains upon infection (Fig. 5C). Specifically, SNRNP40 and SNRNP70 retained the appearance of nuclear speckles following infection, while WDR12 retained a diffuse nuclear distribution. Given that HSV-1 infection disrupts the homeostatic phase boundary segmentation of the nucleolus (*67*, *68*), this trend may be explained by an ability of VICE domains to specifically capture proteins that become misfolded following disruption of their energetically favorable binding environment upon infection. This concept may help explain the intranuclear relocalization behavior that has been observed for other nucleolar host factors, such as fibrillarin (*69*).

The movement of these ribosome biogenesis factors from the nucleolus to VICE domains suggests that the spatial organization of ribosome biogenesis and translation may be broadly affected during infection. Hence, we sought to expand upon prior work on ribosome remodeling during HSV-1 infection by bringing a focus on the nuclear ribosomal population (*68*, *70*, *71*). To understand how HSV-1 infection affects ribosomal abundance and activity, we first performed polysome analysis in cytoplasmic extracts of uninfected and HSV-1–infected cells. This analysis monitors the relative abundance of four ribosome populations: translationally inactive small (40*S*) and large subunits (60*S*), as well as translationally active monosomes (80*S*) and polysomes. Several prominent changes were observed upon HSV-1 infection. First, as expected given the HSV-1–induced translational shutoff, a substantial drop in absorbance of fractions consisting of free mRNA was evident following infection, resulting in a decrease in the polysome-to-monosome ratio (Fig. 5D). This marked drop in polysome prevalence reflects a decrease in bulk translational rate, yet it may also partly derive from a paucity of ribosomes secondary to a defect in ribosome biogenesis. Unexpectedly, however, a similar decline was also observed in the amplitude of the small subunit absorbance peak, potentially reflecting a defect in its synthesis or stability. Next, we performed a sucrose gradient analysis in nuclear extracts (Fig. 5E). We found that uninfected samples exhibited peaks corresponding to the small and large ribosomal subunits, but not monosomes or polysomes in this translationally inactive cellular compartment. In contrast, infected nuclei displayed an apparent loss of the large subunit, suggesting a defect in the production or stability of this subunit in the nucleus. Together, these findings indicated that numerous nucleolar ribosome biogenesis factors are recruited to VICE domains and that translation state and ribosomal subunit abundances are altered in response to infection.

We next considered whether VICE domains also play a role in the spatial organization of rRNA as a means of influencing ribosome biogenesis. We began by measuring the spatial distribution of nascent RNA during infection by pulsing cells with ethynyl uridine followed by click-based fluorescent detection. In contrast to nascent protein, nascent RNA displayed no correlation with VICE domains (Fig. 5F in fibroblasts and fig. S10 in U2OS cells). Considering that measurement of bulk-RNA synthesis in this way may obscure VICE-associated signals corresponding to specific RNA sequences, we used RNA fluorescence in situ hybridization (FISH) to probe for several rRNA transcripts. We specifically targeted premature sequences that have not been extensively processed and which therefore serve as a readout of where ribosome biogenesis is taking place within the cell (Fig. 5G). Again, we found no spatial colocalization between these rRNA transcripts and VICE domains. Therefore, while premature rRNA and ribosome biogenesis proteins colocalize within the nucleolus in healthy cells, the spatial distribution of rRNA and the protein effectors of ribosome biogenesis becomes segregated following infection, with protein components being sequestered within VICE domains.

### Ribosomal proteins of nascent and mature origin are recruited to VICE domains

**Fig. 6.**
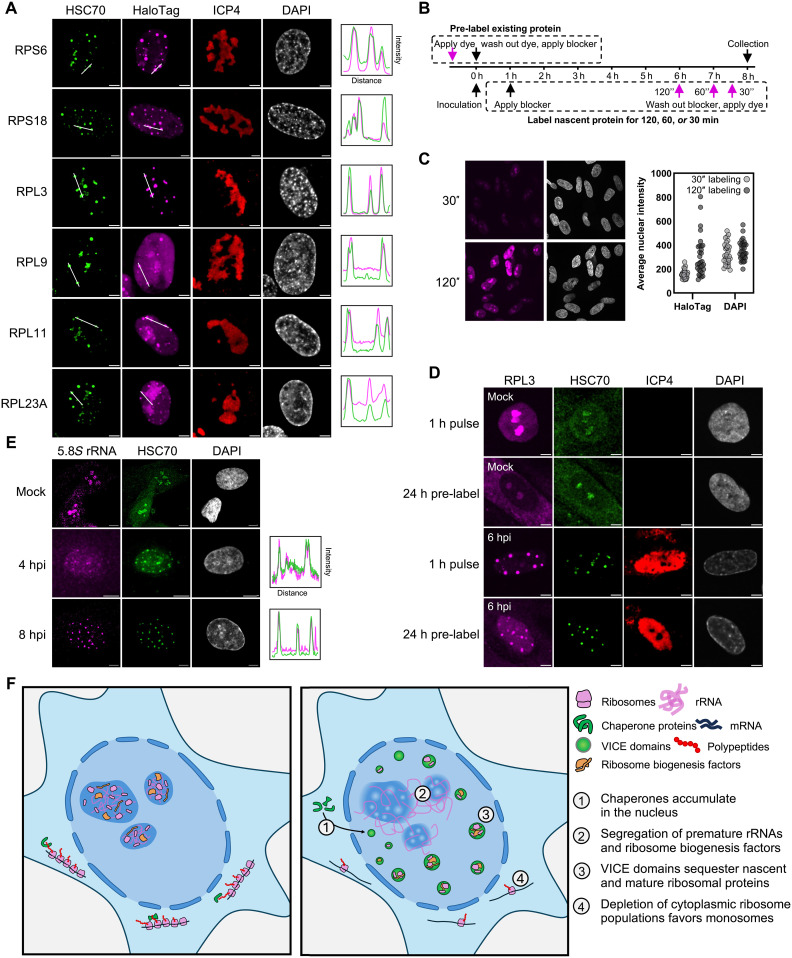
VICE domains recruit both preexisting and newly synthesized ribosomal proteins upon infection. (**A**) HFFs stably expressing the indicated HaloTag fusion protein were mock infected or infected with WT HSV-1 (MOI 5) and collected at 8 hpi, followed by immunofluorescent staining against HSC70 and ICP4. Scale bar, 5 μm. (**B**) Schematic workflow for labeling different molecular populations according to age. Preexisting proteins were specifically labeled by addition of fluorescent HaloTag ligand to the culture media for 30 min, followed by replacement with media containing a nonfluorescent ligand to block further fluorescent incorporation into newly translated populations. Inversely, nascent proteins were specifically labeled by first incubating cells with the nonfluorescent ligand to block fluorescent detection of preexisting populations followed by replacement with media containing fluorescent ligand, allowing only newly translated populations to be labeled. (**C**) HFFs stably expressing RPS6-HaloTag were incubated with fluorescent ligand to label nascent protein for either 120 or 30 min (left), from which nuclear-masked intensities of the ligand versus 4′,6-diamidino-2-phenylindole (DAPI) channels were used to validate time-dependence of fluorescent signal. Scale bar, 10 μm. (**D**) HFFs stably expressing an RPL3 HaloTag fusion construct were treated as described in (B) to differentially label extant and nascent protein populations and collected at 8 hpi, followed by standard immunofluorescent staining against HSC70 and ICP4. Scale bar, 5 μm. (**E**) HFFs were mock infected or infected with WT HSV-1 (MOI 5) and collected at 4 and 8 hpi, followed by immunofluorescent staining against HSC70 and 5.8*S* rRNA. Scale bar, 5 μm. (**F**) Proposed model describing how VICE domains contribute to HSV-1–driven disruption of ribosome biogenesis and translational balance.

In homeostasis, ribosome biogenesis factors and rRNA normally colocalize within the nucleolus to support the maturation and assembly of premature ribosomes. Given that, upon HSV-1 infection, VICE domains sequester ribosome biogenesis factors away from rRNA, we next sought to examine the consequences of infection on ribosomal subunit proteins themselves. Our extended chaperone interactome points to the inclusion of numerous ribosomal subunit proteins within VICE domains (fig. S8). Supporting our interactome studies, we found that all six of the ribosomal subunit proteins we examined colocalized within VICE domains following infection (Fig. 6A).

Because ribosome biogenesis is a pan-cellular process involving many stages, we next sought to define the VICE localization behavior of specific subpopulations of ribosomal proteins. The journey of ribosomal structural proteins throughout the biogenesis process involves considerable dynamic shuttling. Ribosomal protein mRNAs are exported to the cytoplasm where they are translated. Newly synthesized ribosomal proteins are then imported to the nucleus, where they assemble into pre-ribosomal subunits within the nucleolus before being returned to the cytoplasm for final maturation. We therefore aimed to distinguish nascent ribosomal proteins from those belonging to mature ribosomes. Using a dye-based imaging system (*72*), we visualized HaloTag fusion constructs containing our proteins of interest. By pulsing cells at various intervals with either a fluorescent HaloTag ligand (dye) or similar ligand lacking fluorescence (blocker), we were able to track specific subpopulations of individual proteins (Fig. 6B). To label newly synthesized proteins, we cultured cells in media containing the blocker ligand until the addition of the dye. Uninfected cells labeled for 30 min before fixation in this way exhibited substantially lower signal intensity than cells similarly labeled for 120 min, confirming the specific labeling of newly synthesized proteins following removal of the blocker ligand (Fig. 6C). Signal intensity for both populations was also lower than that of samples labeled without the prior addition of blocker, as expected.

To capture the VICE localization behavior of newly synthesized ribosomal proteins, we followed the scheme described above and labeled uninfected or HSV-1–infected samples expressing an RPL3-HaloTag fusion construct for 60 min before fixation at 8 hpi. Signal corresponding to protein synthesized during this 60-min period colocalized with the VICE marker HSC70 and moreover was exclusively nuclear. These results indicate that RPL3 molecules translated following the onset of infection are rapidly localized to VICE domains (Fig. 6D). We then labeled ribosomal proteins extant before infection by culturing cells in media containing dye, which was then washed out and exchanged with media containing blocker ligand 24 hours before infection. This cohort of preexisting RPL3 localized to VICE domains by 8 hpi, although considerable cytoplasmic signal remained. These results suggested that VICE domains recruit not only newly translated ribosomal proteins but also constituents of mature subunits that existed many hours before infection. As an orthogonal validation of this finding, we also observed 5.8*S* rRNA, a processed RNA component found in relatively mature 60*S* (large) subunits, within VICE domains as early as 4 hpi (Fig. 6E). Together, these results highlight how VICE domains exert a sponging effect on critical protein factors involved in the ribosome supply of infected cells.

## DISCUSSION

The dynamic cellular response to viral infection involves the remodeling of normal processes and compartmental boundaries within a host cell as it mounts an emergency response to the presence of the virus. Alterations to the cellular landscape of PPIs following infection are the product of changes in the abundance, stability, posttranslational modification state, and subcellular localization of proteins. Nearly every virus studied is known to disrupt existing organellar structures, as well as to create macromolecular assemblies, both membrane-bound and membraneless. Here, we describe enhancement to a technique capable of performing global protein interaction profiling in dynamic contexts such as viral infection. Specifically, we demonstrate that the accuracy and variety of information gleaned from TPCA is improved substantially when applied to fractionated biological samples. We demonstrate that the thermal stability of most proteins differs substantially depending on their enrichment in either nuclear or cytoplasmic fractions and that by accounting for these differences, N/C-TPCA unlocks information about complex stabilities and PPI predictions that are otherwise obscured in analyses using whole-cell extracts. This was especially true when examining complexes whose assemblies were only evinced in the cellular fraction in which they exhibited lower relative abundance, such as the LAP1-TOR1A and FGFR2-c-Cbl-Lyn-Fyn complexes (fig. S2).

We applied N/C-TPCA to simultaneously capture compartmental protein abundances and PPIs across the replication cycle of a mutant strain of HSV-1 that stimulates a potent innate immune signaling response, yielding a rich resource of protein interactions secondary to infection and immune signaling resolved in time and space. Hybrid analysis of compartmental abundance and PPI dynamics revealed divergent regulation of complexes involved in DNA damage responses upon infection. HSV-1 genomes are decorated by complexes that recognize DNA damage before and during replication, including the RPA complex upstream of ATR and the MRN complex upstream of ataxia-telangiectasia mutated (ATM). An increase in the nuclear abundance of RPA complex components coincided with assembly of this complex in response to infection, consistent with previous findings that HSV-1 preferentially recruits Ataxia telangiectasia and Rad3-related-interacting protein (ATRIP)-RPA to support replication (*57*). In contrast, while the MRN complex exhibited stable assembly in the nucleus throughout infection, its nuclear-to-cytoplasmic abundance ratio markedly decreased as infection progressed. Since ATM and Mre11 are known to exert opposing effects on HSV-1 replication in an ICP0-null context, this finding may represent an ability of HSV-1 to kickstart ATM activation early during infection, followed by relocalization of MRN components to the cytoplasm as a means of evading Mre11-dependent suppression at later time points (*73*). This effect would be similar to a strategy orchestrated during adenovirus infection by the protein E4orf3 (*74*).

By integrating compartmental analysis of abundance dynamics with interaction dynamics, we leveraged our finding that most HSP40, HSP70, and HSP90 family proteins exhibited increased nuclear abundance during infection to expand the known composition of HSV-1–induced nuclear VICE domains. Imaging studies demonstrated the previously unrecognized presence of ribosome biogenesis factors and ribosomal proteins within VICE domains. We also show that premature rRNA is excluded from these bodies, disrupting the status quo colocalization of ribosome biogenesis substrates within the nucleoli of uninfected cells. This spatial decoupling of ribosome biogenesis components was reflected in our observation that the supply of 40*S* and 60*S* ribosomal subunits is markedly reduced in infected cells. Intriguingly, while we demonstrate that newly translated ribosomal proteins are recruited to VICE domains, matching the trend of the bulk proteome as measured by HPG incorporation, recruitment was not limited to this nascent population. Specific labeling of preexisting ribosomal proteins as well as immunofluorescence measurements of 5.8*S* rRNA both exhibited a simultaneous increase in VICE-localized signal and a decrease in cytoplasmic signal upon infection. These findings suggest that the loss of 60*S* and polyribosome signals observed in the cytoplasm following infection may in part be due to relocalization of cytoplasmic ribosome components to the nucleus (Fig. 6F).

These findings fit within the broader context of the evolved strategies mounted by HSV-1 to control the balance of viral versus host gene expression. HSV-1 infection has long been observed to result in translational shutoff. During infection, activation of PKR, which initiates pan-cellular translational shutoff, is inhibited to create room for more tailored viral strategies, such as VHS (UL41). Translation of late viral transcripts is suppressed in the absence of VHS due to the overabundance of early viral transcripts in the ribosome-limited conditions of HSV-1 infection (*75*). Cleavage of early transcripts, which compete for ribosomal docking, therefore, enables late gene expression to proceed. Moreover, HSV-1–infected cells retain translational capacity in the face of ribosomal insufficiency through the activity of VP22, which co-sediments with elongating ribosomes (*76*). HSV-1–infected cells, unlike uninfected cells or those infected with HCMV, retain robust translational capacity following knockdown of most ribosomal proteins (*76*). Our observations that VICE domains impinge upon the supply of both new and extant ribosomal proteins through sequestration of ribosomal structural and biogenesis factors bring a previously unknown perspective to these findings. Together, a broader model arises in which HSV-1 infection creates a ribosome-limiting environment where specially evolved viral factors such as VP22 outcompete the host to maintain translation in a temporal sequence favorable to viral growth and spread.

## MATERIALS AND METHODS

### Prediction of PPIs from TPCA data

PPIs were predicted from TPCA data using Tapioca (*27*), an ensemble machine learning–based method that integrates dynamic MS data, such as from TPCA, with protein physical properties, protein domains, and tissue-specific functional networks (here, a skin-specific functional network was used).

#### Calculating relative z scores

Using the Tapioca prediction two relative *z* scores were calculated for each PPI (one score calculated from each protein within the given PPI) as in Reed *et al.* (*27*). Briefly, for a given PPI consisting of protein 1 and protein 2, the relative *z* score with respect to protein 1 was calculated by first subtracting the mean of all Tapioca scores from PPI pairs containing protein 1 from the Tapioca score of the protein 1–protein 2 PPI. The resulting value was then divided by the SD of all Tapioca scores from PPI pairs containing protein 1, giving the relative *z* score for the PPI of protein 1 and protein 2 with respect to protein 1.

#### Evaluation of PPI predictions

PPI prediction quality was assessed using the gold standard from Reed *et al.* (*27*). This gold standard uses of a refined set of CORUM complexes (*39*) as true-positive examples. Negative examples were generated by taking all pairs of proteins that are not known to localize to the same subcellular compartments, per the human protein atlas database (*37*), and for which no evidence interaction, experimental detected or computationally predicted, appears in the BioGRID (*41*), REACTOME (*43*), or MINT (*42*) databases.

### GO term enrichments

GO term enrichment was performed using one of two tools, HumanBase (*77*) (hb.flatironinstitute.org/) or Enricher (*78*) (maayanlab.cloud/Enrichr/).

### Cytonuclear TPCA MS sample preparation

#### Collection, thermal denaturation, and fractionation

In the cytonuclear TPCA MS experiments, HFFs were first collected via trypsinization and then resuspended in 1× phosphate-buffered saline (PBS). Next, 50 μl of cells was then aliquoted into a strip of polymerase chain reaction (PCR) tubes, with a single PCR strip representing all five temperatures of a single replicate of a single condition. Several additional 50 μl of aliquots were made in a separate strip of PCR tubes to be used as nonthermal denatured reference samples. Thermal denaturation was then performed for 3 min using a thermos cycler (Thermo Fisher Scientific) using five temperatures between 37° and 55° (37°, 40.7°, 44.6°, 52.8°, and 55.3°C) after which the samples were held at 4°C for 3 min. Following thermal denaturation, cytonuclear fractionation was performed using NE-PER nuclear and cytoplasmic extraction reagents (Thermo Fisher Scientific, catalog no. 78833) following the manufacturer’s instructions. Next, 1.5× kinase buffer [75 mM Hepes (pH 7.5), 15 mM MgCl_2_, 1.5× Halt protease and phosphatase inhibitor, and 3 mM tris(2-carboxyethylphosphone) (TCEP)] were added to the isolate nuclear and cytoplasmic fractions. The samples were then snap-frozen and stored at −80°C until preparation for MS analysis.

#### Sample preparation for MS analysis

Thermally denatured samples were thawed on ice and then lysed in 1% Triton X-100, 0.1% Tween-20, and 0.5% sodium deoxycholate in 20 mM Hepes, 110 mM KOAc, 2 mM MgCl_2_, 1 μM ZnCl_2_, and 1 μM CaCl_2_ at pH 7.4 for 1 hour. The samples were then further lysed via three freeze-thaw cycles. Reference samples were lysed in 5% SDS for 1 hour. Thermally denature samples were then centrifuged at 20,000*g* for 20 min at 4°C, pelleting the insoluble protein. The soluble proteins in the supernatant were then transferred to a new 1.5-ml low-bind tube. All samples (thermally denatured and reference) were then reduced and alkylated with 25 mM TCEP and 25 mM chloroacetamide at 55°C for 30 min. Next, methanol-chloroform precipitation was used to purify the protein from the samples. The resulting protein disks were then resuspended in 100 mM Hepes (pH 8.3). Following determination of sample protein concentration by Bicinchoninic Acid (BCA) assay, the samples were aliquoted into new low-bind tubes at a volume giving a concentration of 0.5 mg/ml for the 37°C. The same volume used at 37°C was used for all other temperatures within a given set, giving a concentration typically below 0.5 mg/ml for these samples. The aliquoted samples were then digested at 37°C with 1 μg of sequencing grade trypsin (Thermo Fisher Scientific, catalog no. PI90059) for 16 hours. Next, samples were concentrated to near-dryness in a speed vac before being resuspended in 16 μl of 5% acetonitrile (ACN) in water, followed by labeling by the addition of 95 μg of tandem mass tag (TMT) reagent (TMT 11-plex, Thermo Fisher Scientific, catalog nos. 90406 and A34807). Samples were labeled for 1 hour at room temperature (RT) will shaking at 1000 rpm. Nuclear and cytoplasmic temperatures of the same condition and replicate were multiplexed together (126–131 N, five cytoplasmic fraction temperatures, five nuclear fraction temperatures), along with a reference sample (131C). To generate this reference samples, the previously collected cytoplasmic reference samples across all conditions were combined at an equal volume ratio, and this was repeated for the previously collected nuclear reference samples. These mixed cytoplasmic and nuclear reference samples were then combined in a 1:3 ratio, and this final combined reference sample was labeled (131C). Labeling was stopped by the addition of 0.33% hydroxylamine for (Sigma-Aldrich, catalog no. 467804-10ML) 10 min. To evaluate the quality of labeling, a test mix was generated by combining 1 μl from each labeled sample within the multiplex with 89 μl of 1% trifluoroacetic acid (TFA, Thermo Fisher Scientific, catalog no. 28904). C18 stagetip desalting was performed on the test mix sample before being dried by SpeedVac and then resuspended in 6 μl of 1% formic acid (FA) and 1% ACN. This sample was then analyzed on a Q Exactive HF (QE-HF) MS (Thermo Fisher Scientific). After confirming ≥98% labeling efficiency samples, a quantitative mixture was generated by combining 2 μl from each labeled sample in 300 μl of 0.1% TFA. A Pierce high-pH reversed-phase peptide fractionation column (Thermo Fisher Scientific, catalog no. 84868) was then used to fractionate the mixture into eight fractions. These fractionated samples were then dried down by SpeedVac and then resuspended in 6 μl of 1% FA and 1% ACN before analysis on a QE-HF MS.

### Peptide LC-MS

Liquid chromatography tandem MS (LC-MS/MS) data acquisition and methods generation were performed using Xcalibur v.4 (Thermo Fisher Scientific). For all MS experiments, peptides were resolved for nanoscale LC-MS analysis by a Dionex UltiMate 3000 nRSLC (Thermo Fisher Scientific) equipped with an EASY-Spray C18 column (2-μm particle size, 75-μm diameter, and 500-m length; Thermo Fisher Scientific, catalog no. ES903), using a mixture of solvent A (0.1% FA and 99.9% LC-MS H_2_O) and solvent B (97% LC-MS ACN, 2.9% LC-MS H_2_O, and 0.1% FA). For all MS experiments a QE-HF instrument (Thermo Fisher Scientific) was used.

### TPCA TMT LC-MS/MS analysis

Similar LC-MS/MS settings were used for both the test mix and quant mix samples, with differences noted below. MS/MS spectra were obtained in a full MS/data-dependent MS2 method with full MS operated at 120,000 resolution, a maximum injection time (MIT) of 30 ms, target automatic gain control (AGC) of 3 × 10^6^, and a scan range of 350 to 1800 mass/charge ratio (*m*/*z*). MS/MS scans were performed for the top 20 most intense precursor ions, with a 45,000-resolution, a 72-ms MIT, a target AGC of 1 × 10^5^, a normalized collision energy of 34, and a fixed first mass of 100 *m*/*z*. Dynamic exclusion was used with a 25-s exclusion time.

#### Test mix samples

Peptides were resolved in a linear gradient of 6 to 18% solvent B over 60 min and then 18 to 29% solvent B over 30 min at a flow rate of 250 nl/min. An isolation window of 1.2 *m*/*z* was used.

#### Quant mix samples

Peptides were resolved on a linear gradient of 6 to 18% solvent B for 80 min followed by an 18 to 29% solvent B gradient for 30 min at a flow rate of 250 nl/min. An isolation window of 0.8 *m*/*z* was used.

### Peptide identification and quantification

First, TMT MS/MS spectra were compared to protein sequences of a human reference proteome (downloaded January 2021), an HSV-1 reference proteome (downloaded January 2021) obtained from the UniProt-SwissProt database, and common contaminants using the SEQUEST algorithm in Proteome Discoverer v.2.5 (Thermo Fisher Scientific). A spectral recalibration node was used for the offline recalibration of mass accuracy. Only fully tryptic peptides with a maximum missed cleavage of 2 were included in the database. Cysteine carbamidomethylation (test and quant mix) and TMT labeling on peptide N termini and lysines (quant mix only) were included as static modifications. For dynamic modifications, the same TMT labeling was included for the test mix only. N-terminal methionine loss and acetylation, asparagine deamination, and methionine oxidation were included as dynamic modifications for both the test and quant mixes. A precursor mass tolerance of 4 parts per million (ppm) and fragment ion mass tolerance of 0.02 Da were used. Percolator [false discovery rate (FDR) of 1%] using a reversed sequence database search was used to calculate the FDR of matched spectra. The reporter quantifier node used an integration tolerance of 10 ppm, and the most confident centroid was used for the integration method. In the consensus workflow reporter ions quantifier node, a co-isolation threshold of 30 and an average reporter signal/noise threshold of 8 was used.

### Processing of TPCA data

TPCA data were processed as in Justice *et al.* (*26*) and Reed *et al.* (*27*) with a minor modification. This modification was the scaling of the raw values outputted by Proteome Discoverer v.2.4 using the derived ratios obtained from the test mix. These ratios were calculated from searching the RAW files in Proteome Discoverer v.2.4. and fitting the outputted ratios to a sigmoidal curve to correct for decreased protein solubility at higher temperatures and minimize technical variation between samples. After this scaling, median of median normalization using the reference channel was performed for within plex normalization. Briefly, the median value of all proteins within a single reference channel was calculated for each 11-plex. All proteins across all plexes were divided by the median of these median reference values. Next, cross-plex normalization was performed. For each individual protein, the average reference value of that protein was calculated across all plexes. The resulting value was used to calculate a scaled reference value by dividing an individual protein’s reference channel value in a given plex by the previously calculated average. All values for that given protein were divided by the calculated scaled reference value. Last, the data were fit to a three-parameter log-logistic equation

$$f\left(y\right)=c+\frac{1-c}{1+e^{b\text{[}\text{ln}\left(y\right)-\text{ln}\left(a\right)\text{]}}}$$

### CORUM complex analysis

CORUM complex scores were calculated as in Reed *et al.* (*27*). Briefly, complexes scores were determined by calculating the average Tapioca score for all PPI pairs between subunits of the given complex. To be considered detected and assembled, a CORUM complex had to both have a minimum of 50% of its subunits detected and have a score of greater than or equal to 0.6.

### Calculating protein *T*_m_ and Δ*T*_m_

Protein melting temperature (*T*_m_) and melting temperature shift (Δ*T*_m_) values were calculated as in Lum *et al.* (*79*). Briefly, protein curves were first scanned to identify a temperature at which the protein’s relative abundance was 0.5 ± 0.05. If such a data point was observed, then the associated temperature was set as the protein’s *T*_m_. If not, a custom spline fitting script was used to fit the protein’s melting curve. This fitted curve was then used for interpolation to calculate the temperature associated with a relative abundance of 0.5. If no such temperature existed on the fitted curve, the protein was assigned at *T*_m_ of 55°C, the maximum temperature used for thermal denaturation for TPCA experiments in this study. A protein’s Δ*T*_m_ was calculated by taking the difference between the protein’s *T*_m_ values in two different conditions or subcellular fractions.

### Cell culture conditions

Primary HFF cells (passage numbers 9 to 18) and human embryonic kidney (HEK) 293T cells were cultured in high-glucose Dulbecco’s modified Eagle’s medium (DMEM) (Sigma-Aldrich) supplemented with 10% fetal bovine serum (FBS) (Gemini Bio-Products, 100-106) and 1% (v/v) penicillin-streptomycin solution (Gibco) at 37°C in 5% CO_2_ (standard growth media).

### Virus strains and infections

WT HSV-1 17^+^ (a gift from B. Sodeik, Hannover Medical School, Hannover, Germany), and *ICP0-RF* HSV-1 mutant (a gift from B. Roizman, University of Chicago, Chicago, IL, USA and S. Silverstein, Columbia University, New York, NY, USA) were propagated, aliquoted, snap frozen, and stored at −80°C. Viral strains were expanded and titered in U-2 OS cells. To propagate virus, cells were infected at a low MOI (= 0.001) for 72 to 120 hours until 100% cytopathic effect was observed. Following collection of supernatant, cell-associated virus was liberated from cells by sonication, pooled with the supernatant, and then subjected to ultracentrifugation [20,000 rpm, 2 hours, 4°C with an SW28 swinging bucket rotor (Beckman Coulter)] over a 4% Ficoll cushion to concentrate virus. Stock titers were determined by plaque assay in U-2 OS monolayers.

Infection of HFF cells with ICP0-RF for the main N/C-TPCA dataset was carried out following a synchronous infection protocol described. The cells were precooled by replacing growth medium with CO_2_-independent medium (Gibco, 18045088) containing 0.1% bovine serum albumin (BSA) kept at 4°C and incubated on ice for 15 min. Media were replaced with viral inoculum prepared in the same CO_2_-independent medium/0.1% BSA and incubated on ice in a 4°C environment for 1 hour. The media were then replaced with prewarmed (37°C) DMEM containing 2% FBS and 1% (v/v) penicillin-streptomycin solution and incubated at 37°C for 1 hour. The media were then replaced with ice-cold citrate buffer [40 mM citrate (pH 3), containing 135 mM NaCl, 10 mM KCl, sterile filtered] and incubated for 2 min to inactivate unbound virions. The cells were then washed three times with warm medium and finally replaced with DMEM containing 10% FBS and 1% penicillin-streptomycin until the indicated time point of collection.

All other infections were conducted at the indicated MOI in DMEM supplemented with 2% FBS with intermittent rocking at 37°C and 5% CO_2_. Inoculum was removed after 1 hour, and the media were replaced with standard growth media. In this study, 0 hpi refers to the moment at which inoculum is introduced to the monolayer.

### SDS-PAGE and immunoblotting

To generate thermally denatured samples for Western blot analysis, 2 × 10^6^ cells that had been mock infected or infected with ICP0-RF HSV-1 for 3, 8, or 15 hours were first collected via trypsinization and resuspended in 1× PBS. The cells were then processed through the thermal denaturation protocol described above. Following decantation of the soluble fraction from insoluble pellets, 25% of the original total volume in the nuclear fraction and 10% of the original total volume in the cytoplasmic fraction were prepared for each sample in LDS sample buffer (Invitrogen NP0007) with 2-mercaptoethanol (MilliporeSigma M6250) and boiled at 95°C for 5 min. Gel electrophoresis was performed in 4 to 12% bis-tris gels (Thermo Fisher Scientific, NP0323) using MES SDS running buffer (Thermo Fisher Scientific, B0002). Proteins were then electroblotted onto a 0.45-μm polyvinylidene difluoride membrane (EMD Millipore Immobilon) and blocked with 3% BSA in tris-buffered saline (TBS) for 45 min. Membranes were incubated for 2 hours with primary antibodies diluted into block containing 0.1% Tween (TBST): anti–histone H3 (rabbit, 1:1000; Abcam, ab1791), anti–alpha-tubulin (mouse, 1:1000; Neta Scientific, SIAL-T6199), and anti-ICP4 (mouse, 1:1000; Santa Cruz Biotechnology, sc-69809). After washing three times with TBST, the membranes were incubated for 1 hour at RT with secondary antibodies diluted into block: goat anti-rabbit IgG (H + L) Superclonal recombinant Alexa Fluor 800 (1:10,000; Thermo Fisher Scientific, A27042) and goat anti-mouse IgG (H + L) highly cross-adsorbed Alexa Fluor Plus 680 (1:10,000; Thermo Fisher Scientific, A32730). The blots were washed three times with TBST and imaged on the Odyssey DLx Imaging System (LICORbio).

### Construct and lentivirus generation

HaloTag fusion constructs were generated from PCR fragments amplified off of vectors obtained from the Human ORFeome, Addgene, or DNASU and inserted into an FM5 vector containing standardized linker regions (a gift from D. Sanders) using In-Fusion (Takara Bio). To produce lentivirus from FM5 vectors, HEK293T cells were plated in 6-cm dishes and transfected at ~60% confluency with 1.8 μg of the plasmid containing the fusion gene of interest, 1.4 μg of the psPAX2 lentiviral packaging vector, 0.8 μg of the pMD2.G lentiviral packaging vector, and 12 μl of X-tremeGENE (Sigma-Aldrich) in Opti-MEM (Gibco). The media were replaced at 6 hours post-transfection (hpt) with DMEM supplemented with 20% FBS. The cell culture supernatant was collected at 72 hpt and passed through a 0.45-μm filter to remove cellular debris.

### Lentivirus transductions and plasmid transfections

Lentivirus-containing medium was supplemented with polybrene transfection reagent (8 μg/ml; EMD Millipore) and used to transduce primary HFF cells plated at 60% confluency. The cells were allowed to recover for 2 days before use in experiments or freezing of stocks.

### HaloTag ligands

Fluorescent HaloTag ligands were obtained from Promega (HT1060). The non-fluorescent HaloTag blocking ligand was synthesized as described. Briefly, a 100 mM solution of HaloTag succinimidyl ester (O4) ligand (Promega, P6751) was reacted with 500 mM Tris-HCl buffer (pH 8.0) at 25°C with continuous shaking at 1200 rpm for 120 min to quench the succinidimyl ester moiety.

### RNA Saber FISH

SABER FISH was performed as previously described (*80*). All probe sequences are listed in table S1. Immunofluorescent staining was conducted following FISH, starting from the blocking step. Murine ribonuclease (RNase) inhibitors (NEB, M0314L) were used at a 1:200 dilution throughout the immunofluorescent staining protocol to preserve RNA FISH signals.

### Immunofluorescence microscopy and analysis

HFFs were plated in glass-bottom 96-well plates. Cells were fixed in 4% paraformaldehyde for 15 min at RT, washed with PBS, and then permeabilized in PBS containing 0.1% Triton X-100 (PBT) for 15 min at RT. Blocking was performed with PBT containing 2.5% human serum and 2.5% goat serum for 30 min. The samples were incubated for 2 hours at RT with the primary antibody diluted into block: anti-ICP4 (mouse, 1:500; Santa Cruz Biotechnology, sc-69809) anti-ICP27 (mouse, 1:500; Santa Cruz Biotechnology, sc-69806), anti-PML (rabbit, 1:500, Abcam, ab53773), anti-HSC70 (rat, 1:500, Enzo Life Sciences, 50667581), and anti-5.8*S* rRNA (mouse, VWR, 1:250, 102115-250). After primary staining, the cells were washed three time with PBT and then incubated for 1 hour at RT with 4′,6-diamidino-2-phenylindole (Thermo Fisher Scientific, 1 μg/μl, 62248); the appropriate secondary antibody in block: goat anti-mouse immunoglobulin G (IgG) (H + L) highly cross-adsorbed Alexa Fluor 555 (1:2000; Thermo Fisher Scientific, A28175), goat anti-rat IgG (H + L) highly cross-adsorbed Alexa Fluor 488 (1:2000, Thermo Fisher Scientific, A11006). Plates were imaged at the Princeton Confocal Imaging Core using an inverted fluorescence confocal microscope (W1 SoRa) equipped with a Yokogawa spinning disc (CSU-21) and digital complementary metal-oxide semiconductor camera (Hamamatsu ORCA-Flash TuCam) using a Nikon 60× Plan Apo objective with a ×60 magnification. Image analysis was performed using ImageJ. For determination of colocalization with VICE domains, intensity profiles were measured across a line covering multiple HSC70 aggregates.

### Sucrose gradient fractionation analysis

Cells were passaged approximately 16 hours before harvest, achieving around 70 to 80% confluency the day of the experiment. Before lysis, the cells were pretreated with cycloheximide (100 μg/ml; Thermo Fisher Scientific, AAJ66004XF) in DMEM for 15 min at 37°C.

For cytoplasmic lysate preparation, the cells were washed twice with ice-cold PBS with calcium and magnesium (Thermo Fisher Scientific, 14040182) and lysed on ice using a buffer containing 25 mM Hepes (pH 7.3) (Thermo Fisher Scientific, J16924.AE), 150 mM NaCl (Thermo Fisher Scientific, AM9760G), 15 mM MgCl_2_ (Thermo Fisher Scientific, AM9530G), 8% glycerol (Thermo Fisher Scientific, 327255000), 1% Triton X-100 (Thermo Fisher Scientific, T8787-250ML), 0.5% sodium deoxycholate (Sigma-Aldrich, 30970-100G), cycloheximide (100 μg/ml), 1 mM dithiothreitol (DTT, Promega, P1171), TurboDNase (25 U/ml; Thermo Fisher Scientific, AM2239), SUPERase In RNase inhibitor (100 U/ml; Thermo Fisher Scientific, AM2696), and 1X cOmplete EDTA-free protease inhibitor tablet (Sigma-Aldrich 11836170001) in UltraPure water (Thermo Fisher Scientific, 10977-015). The cells were scraped and harvested in a prechilled tube, in which they were left to lyse for 15 min on ice with frequent pestle douncing. To deplete nuclei, the cells were consecutively centrifuged twice at 800*g*, followed by one centrifugation at 8000*g* and one at 20,000*g*, all for 5 min at 4°C. After this, RNA concentrations were quantified using Qubit RNA Broad Range assay (Thermo Fisher Scientific, Q10210), and normalized amounts of RNA (50 to 100 μg) were layered onto a linear sucrose gradient (10 to 50% sucrose, Thermo Fisher Scientific, AC419762500) prepared with 25 mM Hepes (pH 7.3), 150 mM NaCl, 15 mM MgCl2, cycloheximide (100 μg/ml), and 1 mM DTT in UltraPure water. The gradients were centrifuged in an SW41Ti rotor (Beckman) for 2.5 hours at 40,000 rpm at 4°C. The gradients were passed through a density gradient fractionation system (BioComp) with continuous monitoring of the absorbance at 254 nm.

For nuclear lysate preparation, the cells were washed twice with ice-cold PBS with calcium and magnesium and first harvested on ice into a buffer containing 25 mM Hepes (pH 7.6) (Thermo Fisher Scientific, J61047.AE), 70 mM NaCl, 5 mM MgCl_2_, 70 mM KCl (Thermo Fisher Scientific, AM9640G), 0.3% NP-40 (Sigma-Aldrich, 74385-), 1 mM EDTA (Sigma-Aldrich 324503), 1 mM DTT, and 1X cOmplete EDTA-free protease inhibitor tablet in UltraPure water. The cells were dounced 10 times using an RNase-free pestle (Thermo Fisher Scientific, 12-141-364) and centrifuged for 30 s at 16,000*g*. The supernatant was removed, and the cells were resuspended in the same buffer, repeating the process three times to deplete cytoplasm. The enriched nuclei were then resuspended in a buffer containing 25 mM Hepes (pH 7.6), 10 mM KCl, 10 mM MgCl_2_, 1 mM CaCl_2_ (Sigma-Aldrich 21115), 100 mM arginine (Sigma-Aldrich A5006), 1 mM EDTA, 25 mM adenosine triphosphate (Sigma-Aldrich A7699), 1 mM spermidine (Sigma-Aldrich S0266), 5% glycerol, 0.1% Triton X-100, 1 mM DTT, SUPERase In RNase inhibitor (100 U/ml), TurboDNase (25 U/ml), and 1X cOmplete EDTA-free protease inhibitor tablet in UltraPure water. The nuclei were allowed to lyse on a nutator at 4°C for 30 min. After this, debris was depleted by centrifugation at 20,000*g* for 30 min at 4°C. Then, RNA concentrations were quantified using Qubit RNA Broad Range assay (Thermo Fisher Scientific, Q10210), and normalized amounts of RNA (10 to 30 μg) were layered onto a 10 to 50% linear sucrose gradient and analyzed as above.

### Quantification and statistical analysis

Data processing and large-scale analyses were performed using Python v.3.10.8, using the Python libraries Scipy, Numpy, Pandas, Matplotlib, Seaborn, and Biopython. Cytoscape was used to generate some network visualizations. For boxplots, boxes show median, 25th, and 75th percentile values, with the line within the box representing the median value and whiskers represent ±1.5 interquartile range. Figures were created using Python and Microsoft PowerPoint.

## Supplementary Materials

### The PDF file includes:

Figs. S1 to S10 Table S1 Legends for data files S1 to S6 References
