# Supplementary material for: Sequestration of ribosome biogenesis factors in HSV-1 nuclear aggregates revealed by spatially resolved thermal profiling
Source: Sci Adv. 2025 Jun 27;11(26):eadw6814. doi: 10.1126/sciadv.adw6814 (PMC12204162; doi:10.1126/sciadv.adw6814)
Supplement: Supplementary file 1 — Figs. S1 to S10 Table S1 Legends for data files S1 to S6 References [file sciadv.adw6814_sm.pdf]

Science Advances
AAAS

Supplementary Materials for

Sequestration of ribosome biogenesis factors in HSV-1 nuclear aggregates revealed by spatially resolved thermal profiling

Peter J. Metzger *et al.*

Corresponding author: Ileana M. Cristea, icristea@princeton.edu

Sci. Adv. **11**, eadw6814 (2025)
DOI: 10.1126/sciadv.adw6814

The PDF file includes:

Figs. S1 to S10
Table S1
Legends for data files S1 to S6
References

Other Supplementary Material for this manuscript includes the following:

Data files S1 to S6

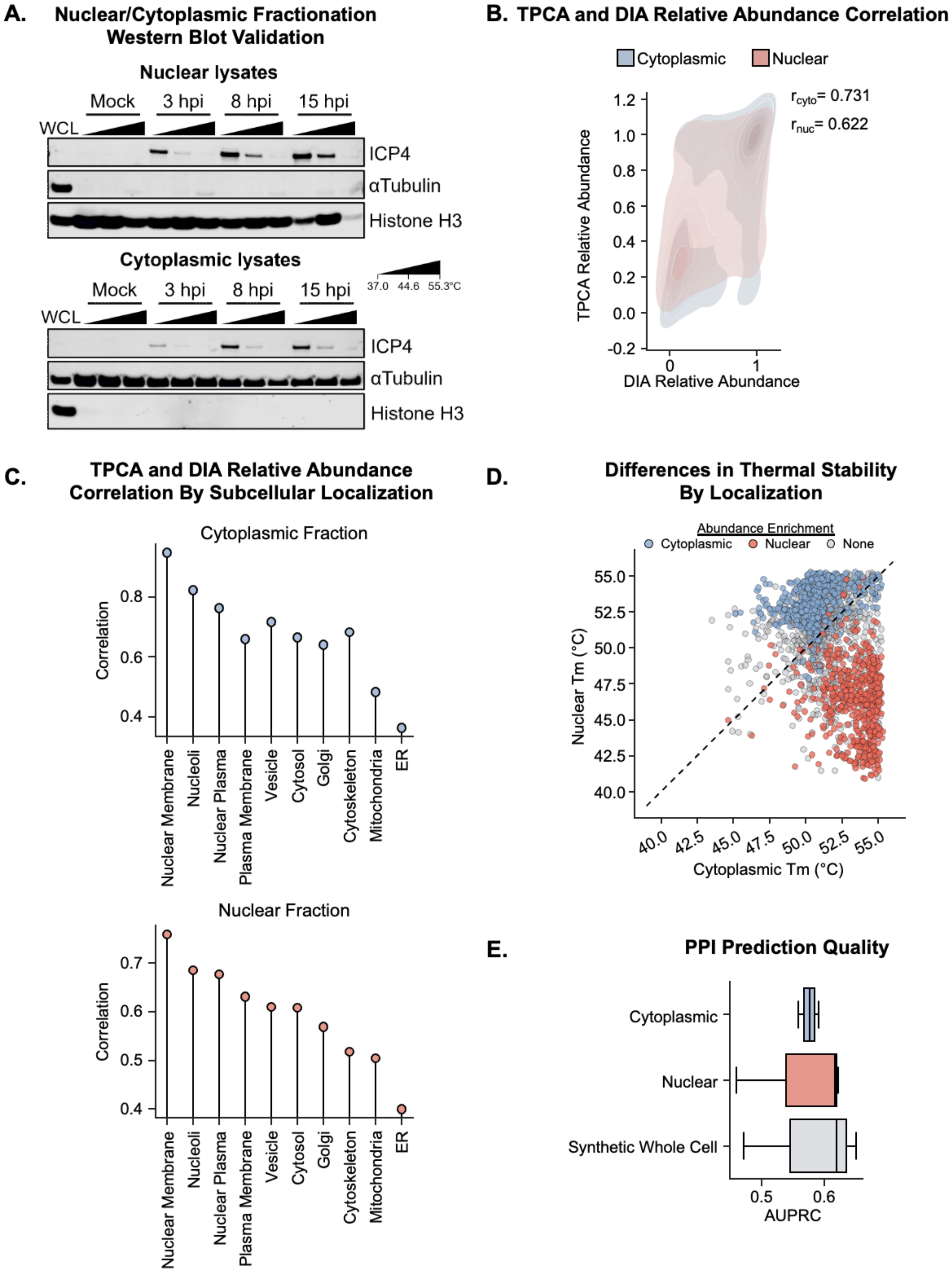

A.
Nuclear/Cytoplasmic Fractionation Western Blot Validation
Nuclear lysates
Mock
3 hpi
8 hpi
15 hpi
WCL
ICP4
αTubulin
Histone H3
Cytoplasmic lysates
37.0
44.6
55.3°C
Mock
3 hpi
8 hpi
15 hpi
WCL
ICP4
αTubulin
Histone H3
B.
TPCA and DIA Relative Abundance Correlation
Cytoplasmic
Nuclear
r_cyto= 0.731
r_nuc= 0.622
TPCA Relative Abundance
1.2
1.0
0.8
0.6
0.4
0.2
0.0
-0.2
0
1
DIA Relative Abundance
C.
TPCA and DIA Relative Abundance Correlation By Subcellular Localization
Cytoplasmic Fraction
Correlation
0.8
0.6
0.4
Nuclear Membrane
Nucleoli
Nuclear Plasma
Plasma Membrane
Vesicle
Cytosol
Golgi
Cytoskeleton
Mitochondria
ER
Nuclear Fraction
Correlation
0.7
0.6
0.5
0.4
Nuclear Membrane
Nucleoli
Nuclear Plasma
Plasma Membrane
Vesicle
Cytosol
Golgi
Cytoskeleton
Mitochondria
ER
D.
Differences in Thermal Stability By Localization
Abundance Enrichment
Cytoplasmic
Nuclear
None
Nuclear Tm (°C)
55.0
52.5
50.0
47.5
45.0
42.5
40.0
40.0
42.5
45.0
47.5
50.0
52.5
55.0
Cytoplasmic Tm (°C)
E.
PPI Prediction Quality
Cytoplasmic
Nuclear
Synthetic Whole Cell
0.5
0.6
AUPRC

Fig S1. Validation of nuclear/cytoplasmic TPCA. (A) Western blot analysis of HFFs subjected to nuclear/cytoplasmic fractionation following either mock infection or infection with ICP0-RF HSV-1 (MOI 5) at 3, 8, and 15 hpi and subjection to a thermal denaturation gradient (37.0, 44.6, 55.3 °C.) Each nuclear sample contains 25% of starting material from this fraction, while each cytoplasmic sample contains 10% of starting material from this fraction. **(B)** The TPCA derived and DIA derived relative abundances of a given protein were compared for all proteins identified in both TPCA and DIA experiment. The fraction-specific Pearson's correlations between the TPCA and DIA values is shown. **(C)** The correlation between TPCA derived and DIA derived relative abundances of protein per fraction (i.e., cytoplasmic or nuclear) per subcellular localization (protein subcellular localizations were obtained from the human protein atlas (*81, 82*). **(D)** Melting temperature (Tm) of proteins cytoplasmic and nuclear fractions. Each dot represents the average Tm value across three replicates for a single protein. Proteins determined (by volcano plot analysis in Fig. 1D) to be significantly enriched in the cytoplasmic fraction are colored blue, while those enriched in the nuclear fraction are colored red. Proteins that were not significantly enriched in either fraction are colored grey. **(E)** AUPRC scores from gold standard evaluation (using gold standard from (*36*)) of Tapioca PPI predictions on cytoplasmic, nuclear, and synthetic whole cell TPCA data. In boxplots, boxes show median, 25th and 75th percentile values, with the line within the box representing the median value, and whiskers represent +/- 1.5 interquartile range.

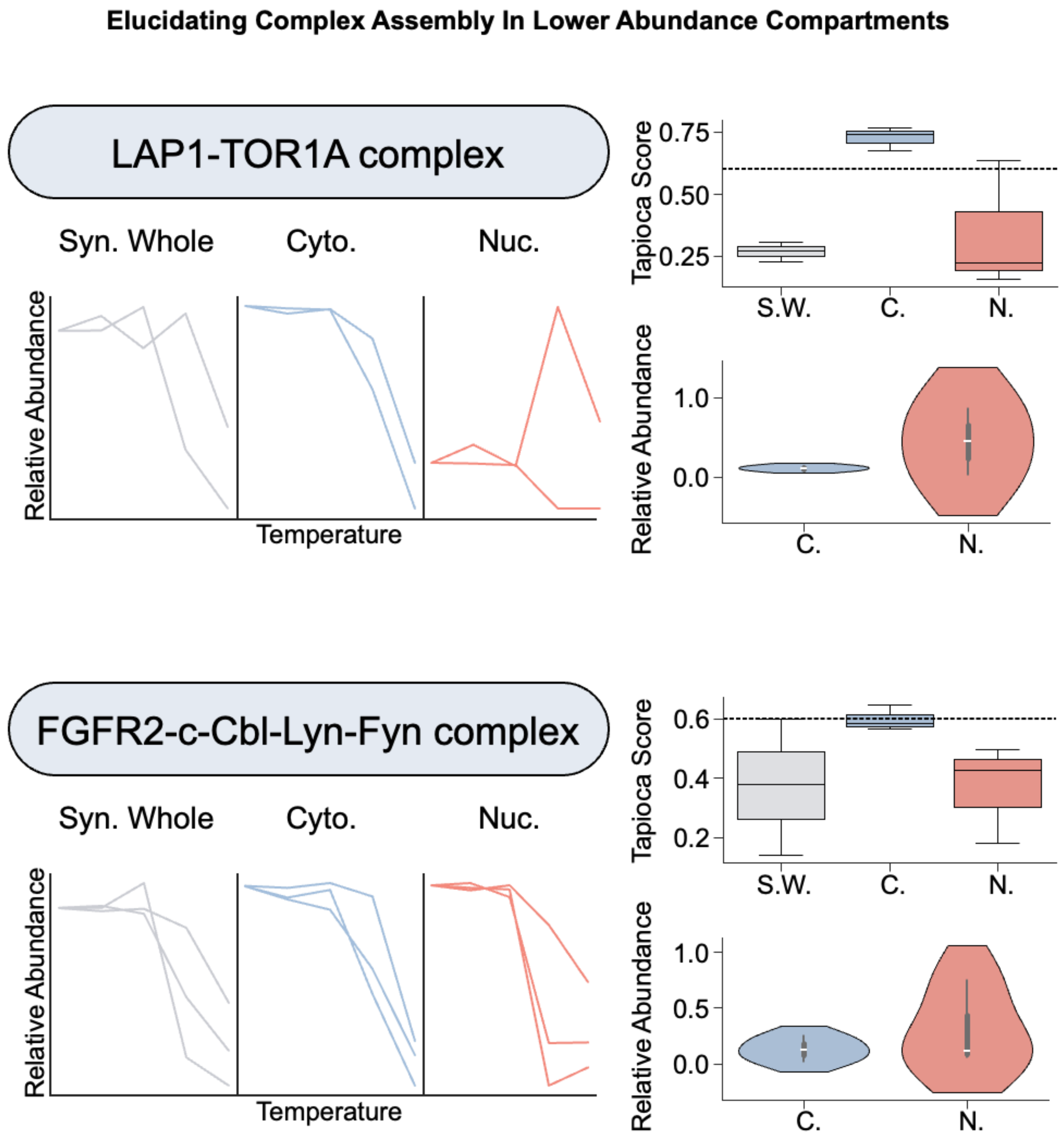

Figure S2. Fractionation enables the detection of protein complex assembly previously obscured by whole cell analysis. Examples of proteins whose assembly is only detectable due to subcellular fractionation. For these vignettes, a synthetic whole cell (Syn. Whole, S.W.) fraction was created by summing the reference normalized cytoplasmic (Cyto., C.) and nuclear (Nuc., N.) melting curves prior to further normalization. In the melting curve plots, each line represents the replicate-averaged melting profile for a single protein within the CORUM complex. CORUM complexes were predicted to be assembled if they achieved an average score greater than or equal to 0.6. This cutoff is shown as a horizontal dotted line in the boxplots. In boxplots, boxes show median, 25th and 75th percentile values, with the line within the box

representing the median value, and whiskers represent +/- 1.5 interquartile range. The reference normalized value at the 37°C for each protein within the complex was used to generate the relative abundance violin plots. For violin plots, the white dot represents the median, the thick black bar represents the ±1.5 interquartile range and the thin gray line represents the total range, excluding outliers. For all experiments depicted, n = 3 biological replicates for each temperature.

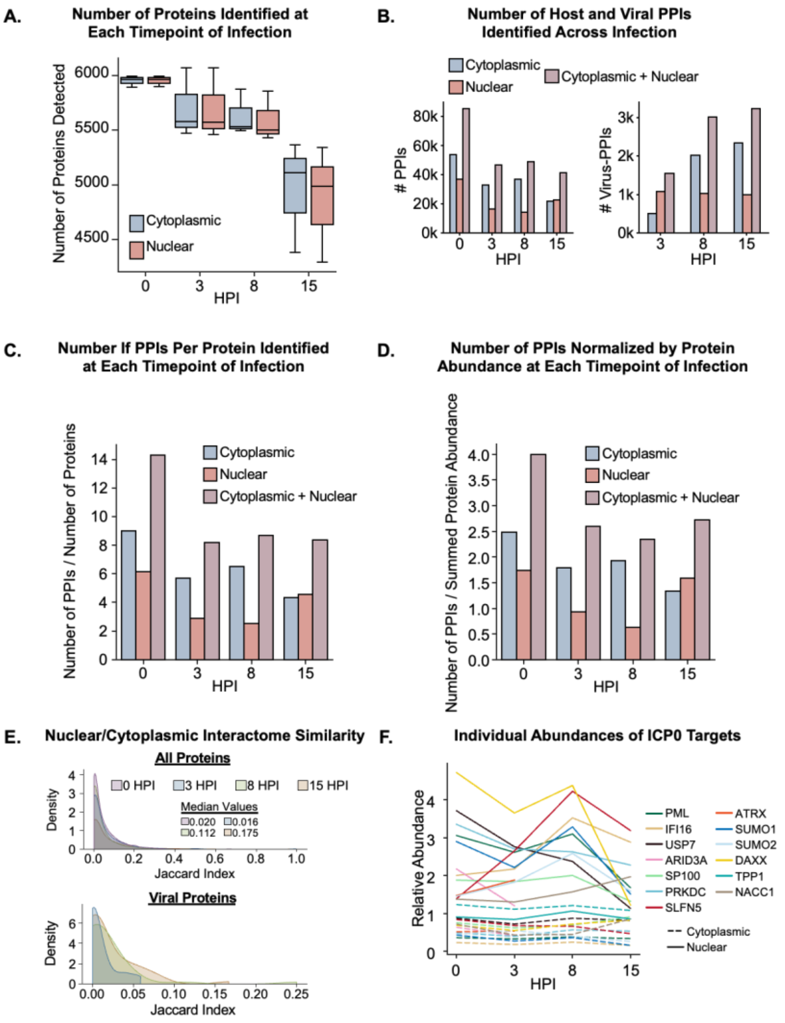

Fig S3. Number of proteins and PPIs and overlap of nuclear and cytoplasmic PPIs detected per infection timepoint, and temporal abundances of ICP0 targets. (A) Number of proteins detected in the nuclear and cytoplasmic fractions per timepoint of infection. In boxplots, boxes show median, 25th and 75th percentile values, with the line within the box representing the median value, and whiskers represent +/- 1.5 interquartile range. **(B)** On the left, number of PPIs predicted in the nuclear, cytoplasmic, and both fractions across per timepoint of infection. On the right, the number of viral PPIs (virus-virus and virus-host) predicted in the nuclear, cytoplasmic, and both fractions across per timepoint of infection. **(C)** Number of PPIs normalized total number of detected proteins per timepoint of infection. **(D)** Number of PPIs normalized by protein abundance per timepoint of infection. **(E)** The cytoplasmic and nuclear interactomes of each protein was compared using the Jaccard index as a measure of similarity (a value of 1 means the interactomes are identical, a value of 0 means they have no overlapping PPIs). The distribution of these Jaccard index values was plotted for each timepoint for all proteins (top chart) and for only viral proteins (bottom chart). **(F)** The nuclear and cytoplasmic abundances throughout infection of host proteins known to be targeted by the viral protein ICP0.

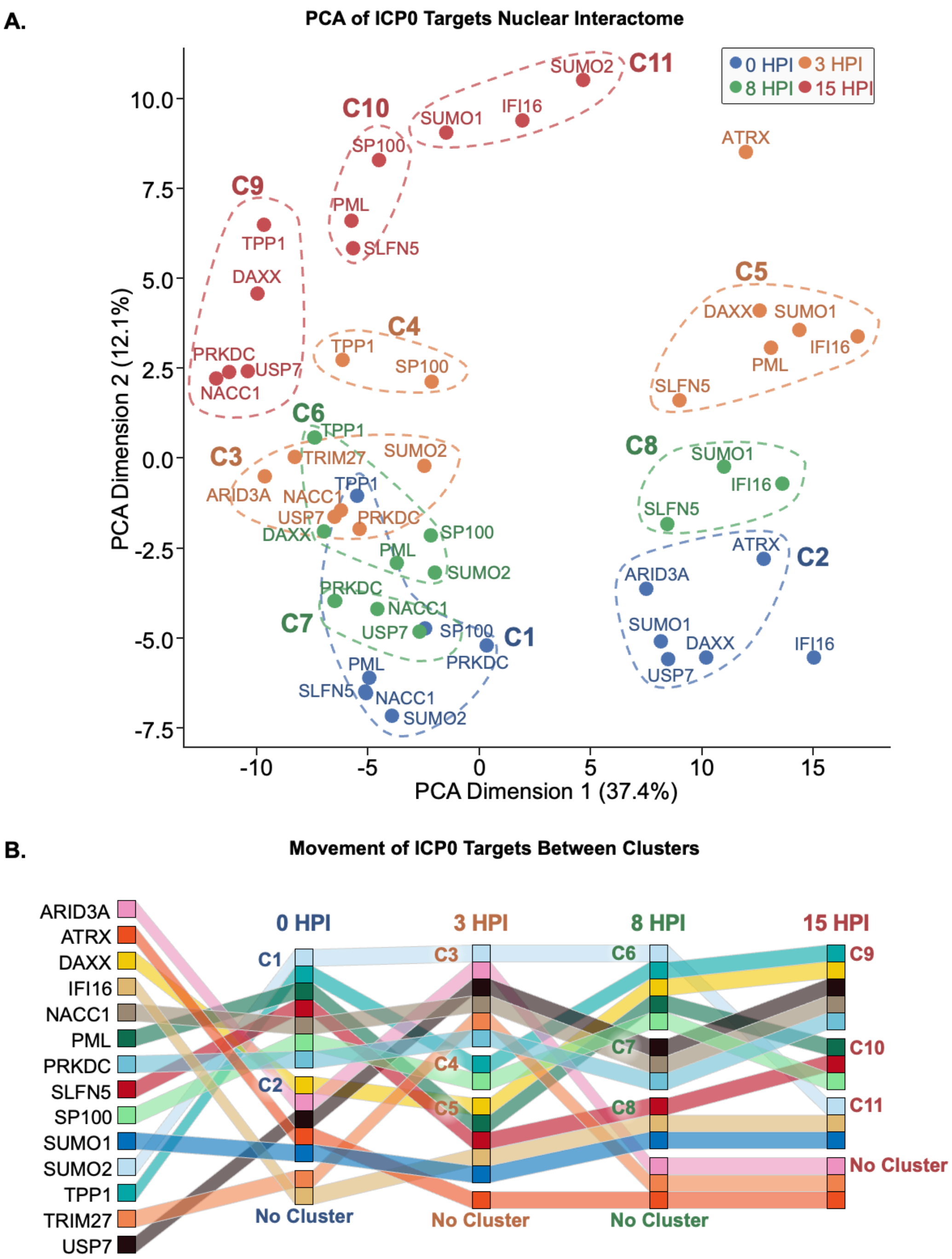

Fig S4. Principle component analysis (PCA) of the interactomes of ICP0 host targets throughout infection. (A) Scatter plot of the values of ICP0 host targets after PCA was performed on their interactomes at each timepoint of infection in which the given target protein was detected. Per timepoint, K-means clustering was performed using either K=3 or K=4. The resulting clusters, that contained more than one protein, are shown by dotted lines and labeled as CX. **(B)** Alluvial plot showing which proteins belong to which clusters at each timepoint of infection. Proteins that clustered by themselves, or where not detected at the given timepoint are assigned to "No Cluster".

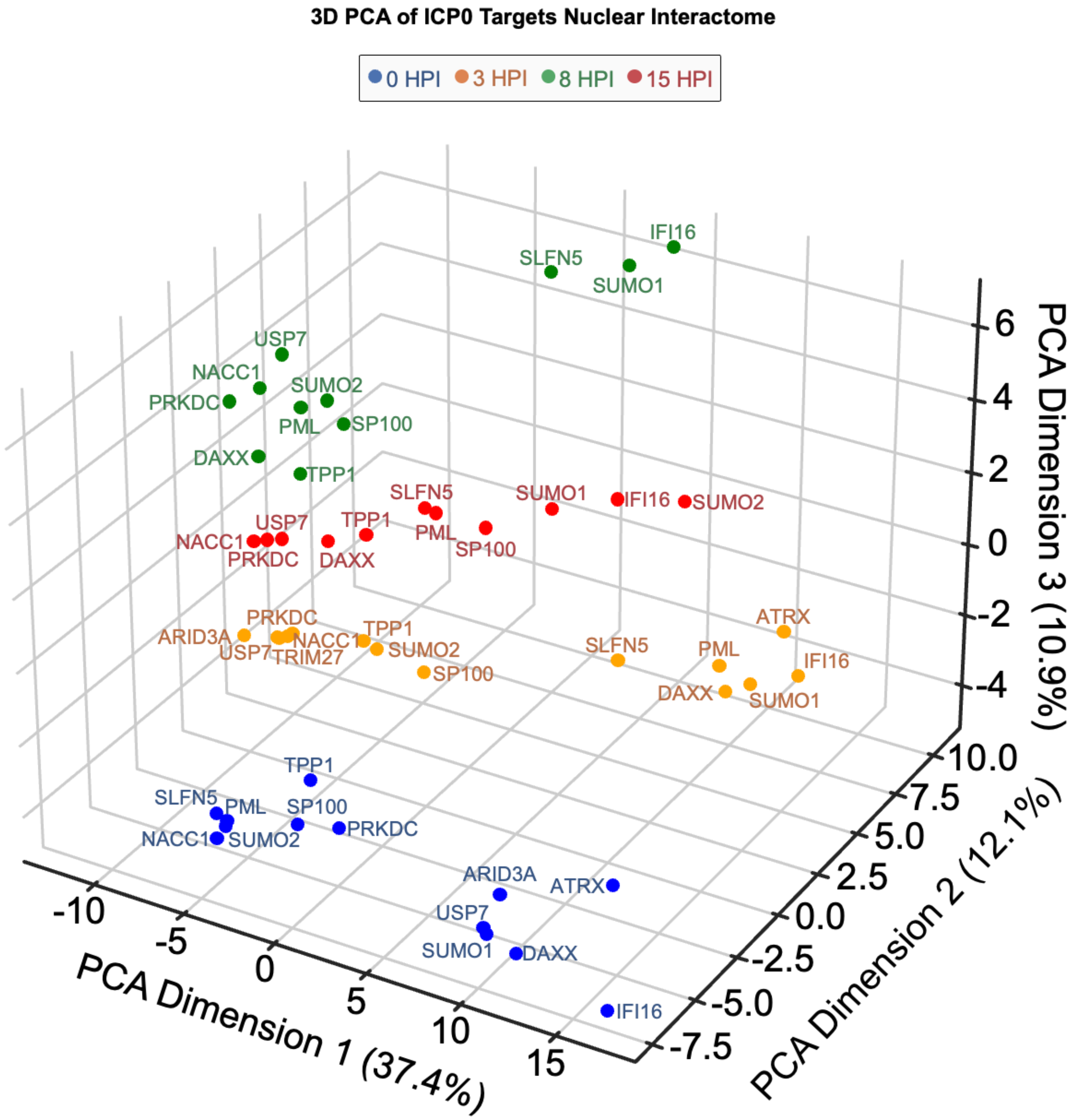

Fig S5. 3D Principle component analysis (PCA) of the interactomes of ICP0 host targets throughout infection. Scatter plot of the values of ICP0 host targets after PCA was performed on their interactomes at each timepoint of infection in which the given target protein was detected. Same as Fig S4A but including the third PCA dimension on the z-axis.

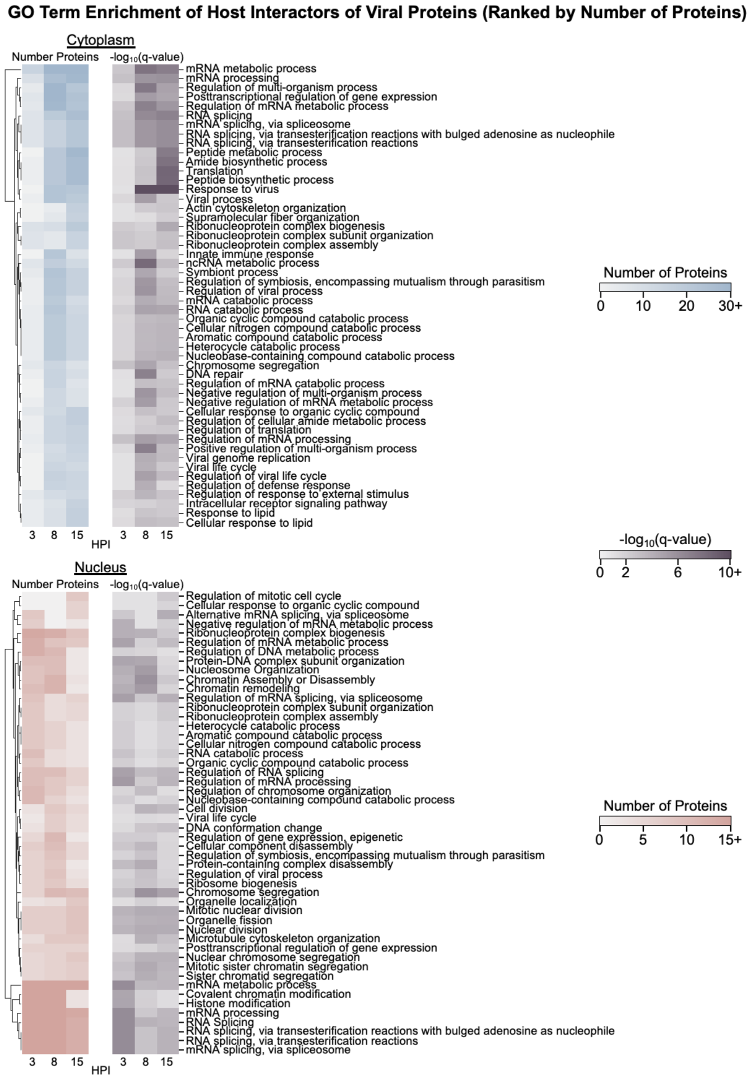

Fig S6. Temporal GO term enrichment of host interactors of viral proteins (Ranked by number of associated proteins). GO term enrichment was performed using HumanBase on the set of host proteins predicted to interact with at least one viral protein throughout the infection time course. These GO terms were then ranked by the maximum number of proteins associated with the given term across all timepoints of infection (cytoplasmic and nuclear fractions were ranked separately). The top 50 GO terms were used to generate a clustermap (a clustered heatmap), clustered by number of proteins associated with the GO term. The top clustermap shows the temporal GO term enrichment of host interactors of viral proteins in the cytoplasmic fractions. The bottom clustermap shows the temporal GO term enrichment of host interactors of viral proteins in the nuclear fractions. The blue and red colored columns represent the number of proteins associated with a given term at a given timepoint in the cytoplasmic and nuclear fractions respectively. The purple columns represent the q-value associated with the term at the given timepoint.

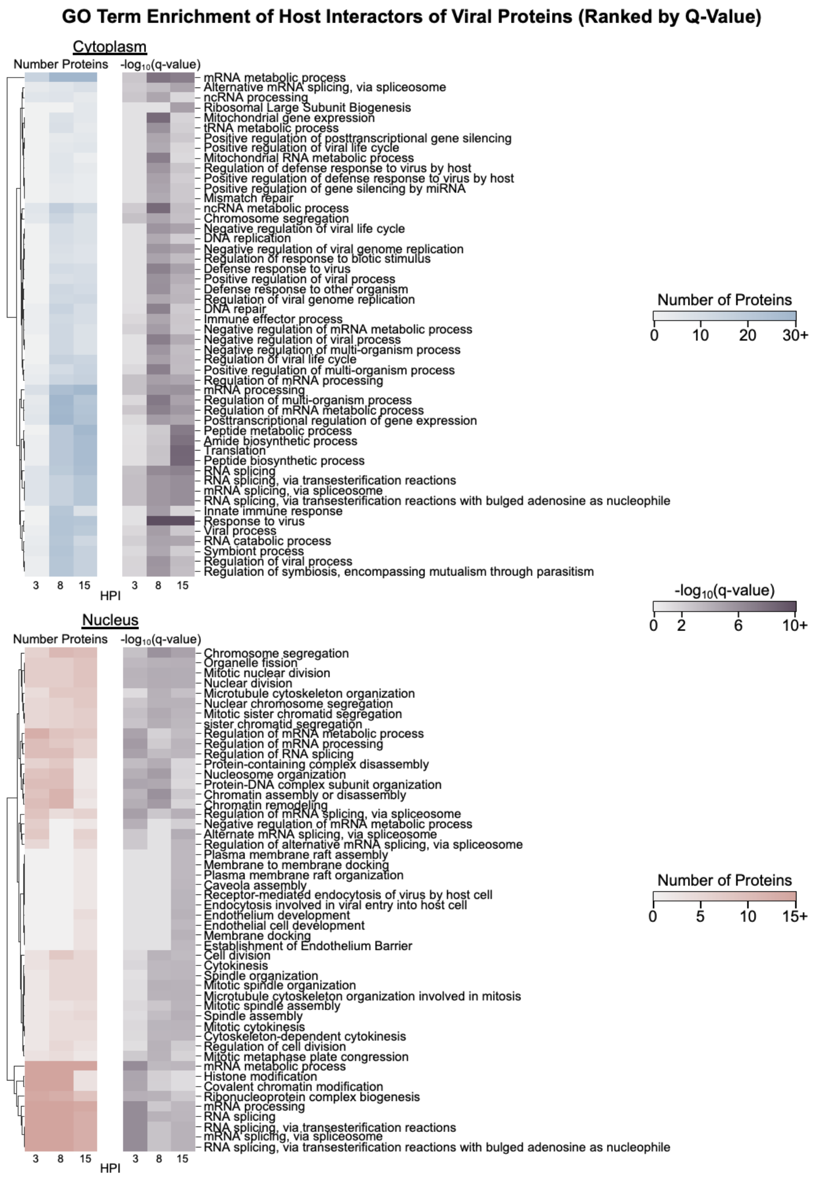

Fig S7. Temporal GO term enrichment of host interactors of viral proteins (Ranked by q-value). GO term enrichment was performed (using HumanBase) (*77*) on the set of host proteins predicted to interact with at least one viral protein throughout the infection time course. These GO terms were then ranked by the minimum q-value across all timepoints of infection (cytoplasmic and nuclear fractions were ranked separately). The top 50 GO terms were used to generate a clustermap (a clustered heatmap), clustered by number of proteins associated with the GO term. The top clustermap shows the temporal GO term enrichment of host interactors of viral proteins in the cytoplasmic fractions. The bottom clustermap shows the temporal GO term enrichment of host interactors of viral proteins in the nuclear fractions. The blue and red colored columns represent the number of proteins associated with a given term at a given timepoint in the cytoplasmic and nuclear fractions respectively. The purple columns represent the q-value associated with the term at the given timepoint.

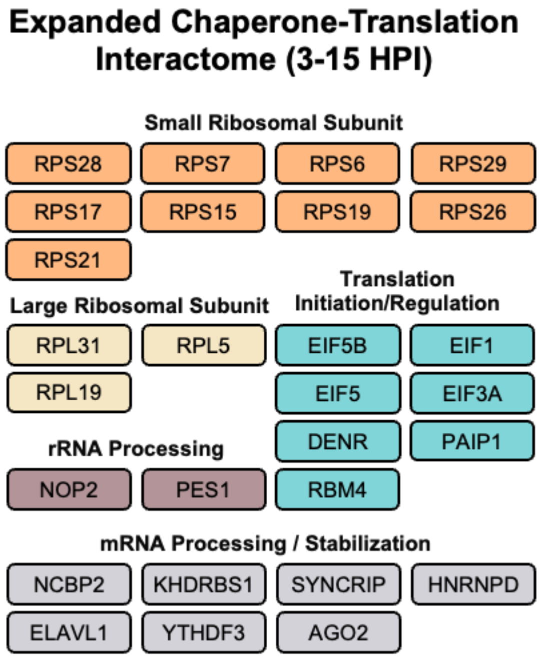

Fig S8. Translation and splicing related proteins that interact with nuclear chaperone-viral protein interactome throughout infection. The set of large and small ribosomal subunits, translation initiation and regulation, rRNA processing, and mRNA processing and stabilization proteins that are predicted to interact with the nuclear chaperone-viral protein interactome during at least one timepoint between 3 and 15 hours post infection.

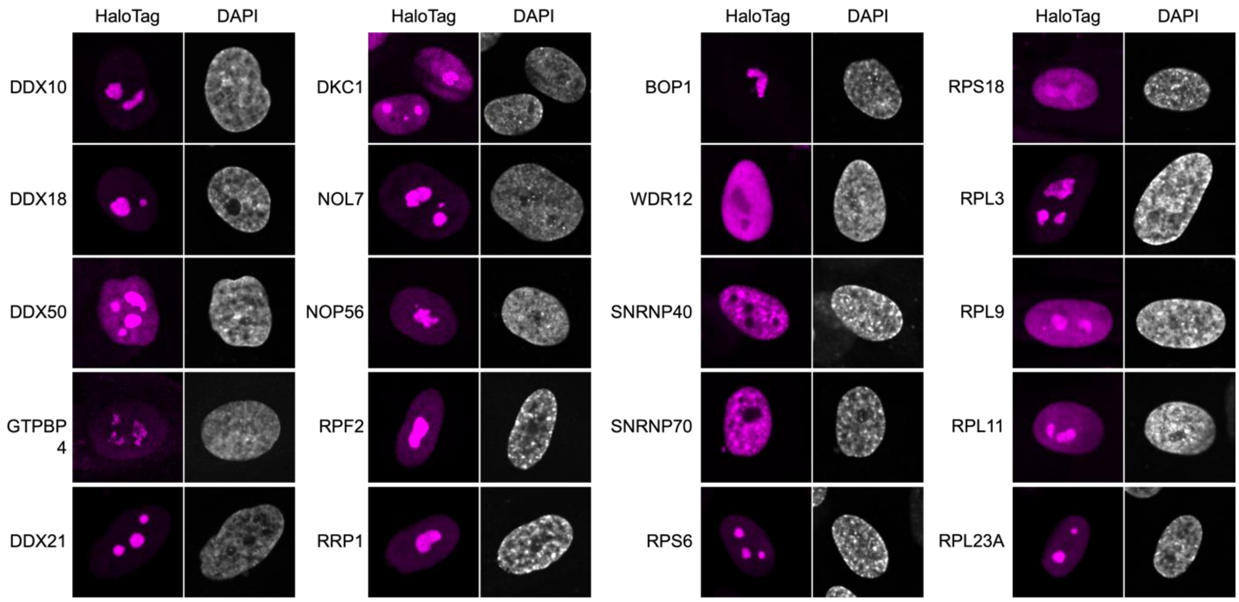

Figure S9. Constructs encoding HaloTag fusion constructs of ribosome biogenesis, splicing, and structural proteins display expected subnuclear localization. HFFs stably expressing the indicated HaloTag fusion construct were analyzed by confocal microscopy. Protein expression patterns were compared to results found in the Human Protein Atlas and the OpenCell project (*81, 82, 83*). Ribosomal proteins and ribosome biogenesis factors displayed expected nucleolar enrichment within the nucleolus, while WDR12 exhibited nucleoplasmic localization and SNRNP40/SNRNP70 each displayed a distribution with the appearance of nuclear speckles.

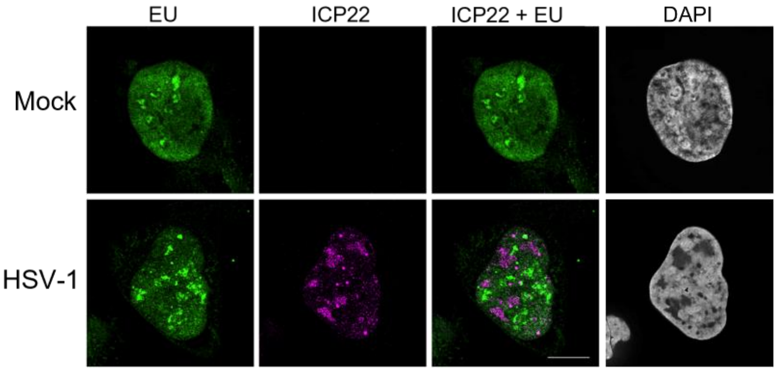

Figure S10. VICE domain localization is spatially distinct from bulk RNA synthesis. U2OS cells were mock infected or infected with ICP22-Flag HSV-1 (MOI 5) and collected at 6 hpi. Thirty minutes prior to collection, 5-ethynyl uridine was added to the media to label nascent RNA, after which sequential click-based fluorescent conjugation and immunofluorescent staining was performed against Flag.

Table S1. RNA FISH probe sequences.

45S	ACGACGTCACCACATCGATCACGAAGAG
Junction 1	ATCAACCAGGTAGGTAAGGTAGAGCGCG
Junction 2	GACCGGACTCCGGAGAGGGGTCGGAAGG
Junction 3	GCTCCGTCGGGAGACGGGCCCGGCGAGG
Junction 4	GGCGATTGATCGGCAAGCGACGCTCAGA

Sequences of RNA FISH probes used in Figure 5G (*84*).

Data S1. (Separate file)
GO term enrichment, from HumanBase, of host proteins interacting with a select set of chaperone / heat shock proteins across timepoints of infection.

Data S2. (Separate file)
Proteins and associated data for nuclear / cytoplasmic ratio volcano plot and associated GO Term enrichment tables from HumanBase. Also, data nuclear / cytoplasmic ratios at an infection timepoint compared to 0 HPI volcano plots.

Data S3. (Separate file)
Cytoplasmic and Nuclear melting point temperatures of proteins.

Data S4. (Separate File)

Compartment-specific Tapioca scores (with a score of 1.0 reflecting the highest confidence of assembly) are listed for complexes annotated in the CORUM database for which at least 50% of complex members were quantified by mass spectrometry. Scores for uninfected cells as well as 3, 8, and 15 hpi are listed.

Data S5. (Separate file)
List of proteins that commonly interact with all ICP0 host protein targets within a given PCA and K-means produced cluster.

Data S6. (Separate file)
GO term enrichment, from HumanBase, of host proteins interacting with viral proteins in cytoplasmic and nuclear fractions across timepoints of infection.

REFERENCES AND NOTES

1. K. Monier, J. C. Armas, S. Etteldorf, P. Ghazal, K. F. Sullivan, Annexation of the interchromosomal space during viral infection. *Nat. Cell Biol.* **2**, 661–665 (2000).

2. H. C. Lewis, L. E. Kelnhofer-Millevolte, M. R. Brinkley, H. E. Arbach, E. A. Arnold, S. Sanders, J. B. Bosse, S. Ramachandran, D. C. Avgousti, HSV-1 exploits host heterochromatin for nuclear egress. *J. Cell Biol.* **222**, e202304106 (2023).

3. F. Maeda, J. Arii, Y. Hirohata, Y. Maruzuru, N. Koyanagi, A. Kato, Y. Kawaguchi, Herpes simplex virus 1 UL34 protein regulates the global architecture of the endoplasmic reticulum in infected cells. *J. Virol.* **91**, e00271–17 (2017).

4. M. C. Rodríguez, J. M. Dybas, J. Hughes, M. D. Weitzman, C. Boutell, The HSV-1 ubiquitin ligase ICP0: Modifying the cellular proteome to promote infection. *Virus Res.* **285**, 198015 (2020).

5. M. K. Chelbi-Alix, H. de Thé, Herpes virus induced proteasome-dependent degradation of the nuclear bodies-associated PML and Sp100 proteins. *Oncogene* **18**, 935–941 (1999).

6. C. Boutell, S. Sadis, R. D. Everett, Herpes simplex virus type 1 immediate-early protein ICP0 and is isolated RING finger domain act as ubiquitin E3 ligases in vitro. *J. Virol.* **76**, 841–850 (2002).

7. R. D. Everett, G. G. Maul, HSV-1 IE protein Vmw110 causes redistribution of PML. *EMBO J.* **13**, 5062–5069 (1994).

8. S. Müller, A. Dejean, Viral immediate-early proteins abrogate the modification by SUMO-1 of PML and Sp100 proteins, correlating with nuclear body disruption. *J. Virol.* **73**, 5137–5143 (1999).

9. J. Parkinson, R. D. Everett, Alphaherpesvirus proteins related to herpes simplex virus type 1 ICP0 affect cellular structures and proteins. *J. Virol.* **74**, 10006–10017 (2000).

10. T. Alandijany, A. P. E. Roberts, K. L. Conn, C. Loney, S. McFarlane, A. Orr, C. Boutell, Distinct temporal roles for the promyelocytic leukaemia (PML) protein in the sequential regulation of intracellular host immunity to HSV-1 infection. *PLOS Pathog.* **14**, e1006769 (2018).

11. H. Dong, W. Wu, J. Li, Y. Ma, X. Deng, D. Guo, P. Xu, PML body component Sp100A is a cytosolic responder to IFN and activator of antiviral ISGs. *MBio* **13**, e0204422 (2022).

12. A. V. Chee, P. Lopez, P. P. Pandolfi, B. Roizman, Promyelocytic leukemia protein mediates interferon-based anti-herpes simplex virus 1 effects. *J. Virol.* **77**, 7101–7105 (2003).

13. B. Dauber, D. Poon, T. Dos Santos, B. A. Duguay, N. Mehta, H. A. Saffran, J. R. Smiley, The herpes simplex virus virion host shutoff protein enhances translation of viral true late mRNAs independently of suppressing protein kinase R and stress granule formation. *J. Virol.* **90**, 6049–6057 (2016).

14. A. Esclatine, B. Taddeo, B. Roizman, Herpes simplex virus 1 induces cytoplasmic accumulation of TIA-1/TIAR and both synthesis and cytoplasmic accumulation of tristetraprolin, two cellular proteins that bind and destabilize AU-rich RNAs. *J. Virol.* **78**, 8582–8592 (2004).

15. A. Esclatine, B. Taddeo, B. Roizman, The UL41 protein of herpes simplex virus mediates selective stabilization or degradation of cellular mRNAs. *Proc. Natl. Acad. Sci. U.S.A.* **101**, 18165–18170 (2004).

16. B. Taddeo, W. Zhang, B. Roizman, The herpes simplex virus host shutoff RNase degrades cellular and viral mRNAs made before infection but not viral mRNA made after infection. *J. Virol.* **87**, 4516–4522 (2013).

17. P. Feng, D. N. Everly, G. S. Read, mRNA Decay during herpesvirus infections: Interaction between a putative viral nuclease and a cellular translation factor. *J. Virol.* **75**, 10272–10280 (2001).

18. C. Salaun, A. I. MacDonald, O. Larralde, L. Howard, K. Lochtie, H. M. Burgess, M. Brook, P. Malik, N. K. Gray, S. V. Graham, Poly(A)-binding protein 1 partially relocalizes to the nucleus

during herpes simplex virus type 1 infection in an ICP27-independent manner and does not inhibit virus replication. *J. Virol.* **84**, 8539–8548 (2010).

19. G. Elliott, K. Pheasant, K. Ebert-Keel, J. Stylianou, A. Franklyn, J. Jones, Multiple posttranscriptional strategies to regulate the herpes simplex virus 1 vhs endoribonuclease. *J. Virol.* **92**, e00818–18 (2018).

20. E. Dobrikova, M. Shveygert, R. Walters, M. Gromeier, Herpes simplex virus proteins ICP27 and UL47 associate with polyadenylate-binding protein and control its subcellular distribution. *J. Virol.* **84**, 270–279 (2010).

21. T. M. Greco, M. A. Kennedy, I. M. Cristea, Proteomic technologies for deciphering local and global protein interactions. *Trends Biochem. Sci.* **45**, 454–455 (2020).

22. C. D. Go, J. D. R. Knight, A. Rajasekharan, B. Rathod, G. G. Hesketh, K. T. Abe, J.-Y. Youn, P. Samavarchi-Tehrani, H. Zhang, L. Y. Zhu, E. Popiel, J.-P. Lambert, É. Coyaud, S. W. T. Cheung, D. Rajendran, C. J. Wong, H. Antonicka, L. Pelletier, A. F. Palazzo, E. A. Shoubridge, B. Raught, A.-C. Gingras, A proximity-dependent biotinylation map of a human cell. *Nature* **595**, 120–124 (2021).

23. M. M. Savitski, F. B. M. Reinhard, H. Franken, T. Werner, M. F. Savitski, D. Eberhard, D. M. Molina, R. Jafari, R. B. Dovega, S. Klaeger, B. Kuster, P. Nordlund, M. Bantscheff, G. Drewes, Tracking cancer drugs in living cells by thermal profiling of the proteome. *Science* **346**, 1255784 (2014).

24. A. Mateus, N. Kurzawa, I. Becher, S. Sridharan, D. Helm, F. Stein, A. Typas, M. M. Savitski, Thermal proteome profiling for interrogating protein interactions. *Mol. Syst. Biol.* **16**, e9232 (2020).

25. Y. Hashimoto, X. Sheng, L. A. Murray-Nerger, I. M. Cristea, Temporal dynamics of protein complex formation and dissociation during human cytomegalovirus infection. *Nat. Commun.* **11**, 806 (2020).

26. J. L. Justice, M. A. Kennedy, J. E. Hutton, D. Liu, B. Song, B. Phelan, I. M. Cristea, Systematic profiling of protein complex dynamics reveals DNA-PK phosphorylation of IFI16 en route to herpesvirus immunity. *Sci. Adv.* **7**, eabg6680 (2021).

27. T. J. Reed, M. D. Tyl, A. Tadych, O. G. Troyanskaya, I. M. Cristea, Tapioca: A platform for predicting de novo protein–protein interactions in dynamic contexts. *Nat. Methods* **21**, 488–500 (2024).

28. J. Selkrig, M. Stanifer, A. Mateus, K. Mitosch, I. Barrio-Hernandez, M. Rettel, H. Kim, C. G. P. Voogdt, P. Walch, C. Kee, N. Kurzawa, F. Stein, C. Potel, A. Jarzab, B. Kuster, R. Bartenschlager, S. Boulant, P. Beltrao, A. Typas, M. M. Savitski, SARS-CoV-2 infection remodels the host protein thermal stability landscape. *Mol. Syst. Biol.* **17**, e10188 (2021).

29. C. S. H. Tan, K. D. Go, X. Bisteau, L. Dai, C. H. Yong, N. Prabhu, M. B. Ozturk, Y. T. Lim, L. Sreekumar, J. Lengqvist, V. Tergaonkar, P. Kaldis, R. M. Sobota, P. Nordlund, Thermal proximity coaggregation for system-wide profiling of protein complex dynamics in cells. *Science* **359**, 1170–1177 (2018).

30. E. K. Lium, S. Silverstein, Mutational analysis of the herpes simplex virus type 1 ICP0 C3HC4 zinc ring finger reveals a requirement for ICP0 in the expression of the essential alpha27 gene. *J. Virol.* **71**, 8602–8614 (1997).

31. B. A. Diner, K. K. Lum, A. Javitt, I. M. Cristea, Interactions of the antiviral factor interferon Gamma-inducible protein 16 (IFI16) mediate immune signaling and herpes simplex virus-1 immunosuppression. *Mol. Cell. Proteomics* **14**, 2341–2356 (2015).

32. C. M. Livingston, M. F. Ifrim, A. E. Cowan, S. K. Weller, Virus-induced chaperone-enriched (VICE) domains function as nuclear protein quality control centers during HSV-1 infection. *PLOS Pathog.* **5**, e1000619 (2009).

33. A. D. Burch, S. K. Weller, Nuclear sequestration of cellular chaperone and proteasomal machinery during herpes simplex virus type 1 infection. *J. Virol.* **78**, 7175–7185 (2004).

34. F.-M. Boisvert, S. van Koningsbruggen, J. Navascués, A. I. Lamond, The multifunctional nucleolus. *Nat. Rev. Mol. Cell Biol.* **8**, 574–585 (2007).

35. K. Suzuki, P. Bose, R. Y. Leong-Quong, D. J. Fujita, K. Riabowol, REAP: A two minute cell fractionation method. *BMC. Res. Notes* **3**, 294 (2010).

36. S. Sun, Z. Zheng, J. Wang, F. Li, A. He, K. Lai, S. Zhang, J.-H. Lu, R. Tian, C. S. H. Tan, Improved in situ characterization of protein complex dynamics at scale with thermal proximity co-aggregation. *Nat. Commun.* **14**, 7697 (2023).

37. The Human Protein Atlas, https://proteinatlas.org/.

38. A. Mateus, T. A. Määttä, M. M. Savitski, Thermal proteome profiling: Unbiased assessment of protein state through heat-induced stability changes. *Proteome Sci.* **15**, 13 (2016).

39. G. Tsitsiridis, R. Steinkamp, M. Giurgiu, B. Brauner, G. Fobo, G. Frishman, C. Montrone, A. Ruepp, CORUM: The comprehensive resource of mammalian protein complexes–2022. *Nucleic Acids Res.* **51**, D539–D545 (2023).

40. R. G. Stacey, M. A. Skinnider, J. H. L. Chik, L. J. Foster, Context-specific interactions in literature-curated protein interaction databases. *BMC Genomics* **19**, 758 (2018).

41. R. Oughtred, J. Rust, C. Chang, B.-J. Breitkreutz, C. Stark, A. Willems, L. Boucher, G. Leung, N. Kolas, F. Zhang, S. Dolma, J. Coulombe-Huntington, A. Chatr-aryamontri, K. Dolinski, M. Tyers, The BioGRID database: A comprehensive biomedical resource of curated protein, genetic, and chemical interactions. *Protein Sci.* **30**, 187–200 (2021).

42. L. Licata, L. Briganti, D. Peluso, L. Perfetto, M. Iannuccelli, E. Galeota, F. Sacco, A. Palma, A. P. Nardozza, E. Santonico, L. Castagnoli, G. Cesareni, MINT, the molecular interaction database: 2012 update. *Nucleic Acids Res.* **40**, D857–D861 (2012).

43. M. Gillespie, B. Jassal, R. Stephan, M. Milacic, K. Rothfels, A. Senff-Ribeiro, J. Griss, C. Sevilla, L. Matthews, C. Gong, C. Deng, T. Varusai, E. Ragueneau, Y. Haider, B. May, V. Shamovsky, J. Weiser, T. Brunson, N. Sanati, L. Beckman, X. Shao, A. Fabregat, K.

Sidiropoulos, J. Murillo, G. Viteri, J. Cook, S. Shorser, G. Bader, E. Demir, C. Sander, R. Haw, G. Wu, L. Stein, H. Hermjakob, P. D'Eustachio, The reactome pathway knowledgebase 2022. *Nucleic Acids Res.* **50**, D687–D692 (2022).

44. H. Gu, B. Roizman, The two functions of herpes simplex virus 1 ICP0, inhibition of silencing by the CoREST/REST/HDAC complex and degradation of PML, are executed in tandem. *J. Virol.* **83**, 181–187 (2009).

45. D. Devadas, T. Koithan, R. Diestel, U. Prank, B. Sodeik, K. Döhner, Herpes simplex virus internalization into epithelial cells requires Na+/H+ exchangers and p21-activated kinases but neither clathrin- nor caveolin-mediated endocytosis. *J. Virol.* **88**, 13378–13395 (2014).

46. R. D. Everett, C. Boutell, A. Orr, Phenotype of a herpes simplex virus type 1 mutant that fails to express immediate-early regulatory protein ICP0. *J. Virol.* **78**, 1763–1774 (2004).

47. E. T. Kim, J. M. Dybas, K. Kulej, E. D. Reyes, A. M. Price, L. N. Akhtar, A. Orr, B. A. Garcia, C. Boutell, M. D. Weitzman, Comparative proteomics identifies Schlafen 5 (SLFN5) as a herpes simplex virus restriction factor that suppresses viral transcription. *Nat. Microbiol.* **6**, 234–245 (2021).

48. R. D. Everett, C. Parada, P. Gripon, H. Sirma, A. Orr, Replication of ICP0-null mutant herpes simplex virus type 1 is restricted by both PML and Sp100. *J. Virol.* **82**, 2661–2672 (2008).

49. R. J. Sydiskis, B. Roizman, Polysomes and protein synthesis in cells infected with a DNA virus. *Science* **153**, 76–78 (1966).

50. J. Huang, H. You, C. Su, Y. Li, S. Chen, C. Zheng, Herpes simplex virus 1 tegument protein VP22 abrogates cGAS/STING-mediated antiviral innate immunity. *J. Virol.* **92**, e00841–18 (2018).

51. G. Xu, C. Liu, S. Zhou, Q. Li, Y. Feng, P. Sun, H. Feng, Y. Gao, J. Zhu, X. Luo, Q. Zhan, S. Liu, S. Zhu, H. Deng, D. Li, P. Gao, Viral tegument proteins restrict cGAS-DNA phase separation to mediate immune evasion. *Mol. Cell* **81**, 2823–2837.e9 (2021).

52. L. A. Murray-Nerger, J. L. Justice, P. Rekapalli, J. E. Hutton, I. M. Cristea, Lamin B1 acetylation slows the G1 to S cell cycle transition through inhibition of DNA repair. *Nucleic Acids Res.* **49**, 2044–2064 (2021).

53. J. B. Bosse, I. B. Hogue, M. Feric, S. Y. Thiberge, B. Sodeik, C. P. Brangwynne, L. W. Enquist, Remodeling nuclear architecture allows efficient transport of herpesvirus capsids by diffusion. *Proc. Natl. Acad. Sci. U.S.A.* **112**, E5725–E5733 (2015).

54. M. H. Christensen, S. B. Jensen, J. J. Miettinen, S. Luecke, T. Prabakaran, L. S. Reinert, T. Mettenleiter, Z. J. Chen, D. M. Knipe, R. M. Sandri-Goldin, L. W. Enquist, R. Hartmann, T. H. Mogensen, S. A. Rice, T. A. Nyman, S. Matikainen, S. R. Paludan, HSV-1 ICP27 targets the TBK1-activated STING signalsome to inhibit virus-induced type I IFN expression. *EMBO J.* **35**, 1385–1399 (2016).

55. C. Boutell, S. Sadis, R. D. Everett, Herpes simplex virus type 1 immediate-early protein ICP0 and its isolated RING finger domain act as ubiquitin E3 ligases in vitro. *J. Virol.* **76**, 841–850 (2002).

56. M. H. Orzalli, N. A. DeLuca, D. M. Knipe, Nuclear IFI16 induction of IRF-3 signaling during herpesviral infection and degradation of IFI16 by the viral ICP0 protein. *Proc. Natl. Acad. Sci. U.S.A.* **109**, E3008–E3017 (2012).

57. K. N. Mohni, A. R. Dee, S. Smith, A. J. Schumacher, S. K. Weller, Efficient herpes simplex virus 1 replication requires cellular ATR pathway proteins. *J. Virol.* **87**, 531–542 (2013).

58. B. Feierbach, S. Piccinotti, M. Bisher, W. Denk, L. W. Enquist, Alpha-herpesvirus infection induces the formation of nuclear actin filaments. *PLOS Pathog.* **2**, e85 (2006).

59. Z. Wang, Y. Li, X. Yang, J. Zhao, Y. Cheng, J. Wang, Mechanism and complex roles of HSC70 in viral infections. *Front. Microbiol.* **11**, 1577 (2020).

60. L. Li, L. A. Johnson, J. Q. Dai-Ju, R. M. Sandri-Goldin, Hsc70 focus formation at the periphery of HSV-1 transcription sites requires ICP27. *PLOS ONE* **3**, e1491 (2008).

61. A. D. Burch, S. K. Weller, Herpes simplex virus type 1 DNA polymerase requires the mammalian chaperone hsp90 for proper localization to the nucleus. *J. Virol.* **79**, 10740–10749 (2005).

62. C. S. H. Teo, R. A. Serwa, P. O'Hare, Spatial and temporal resolution of global protein synthesis during HSV infection using bioorthogonal precursors and click chemistry. *PLOS Pathog.* **12**, e1005927 (2016).

63. X. Shi, Y. Li, H. Zhou, X. Hou, J. Yang, V. Malik, F. Faiola, J. Ding, X. Bao, M. Modic, W. Zhang, L. Chen, S. R. Mahmood, E. Apostolou, F.-C. Yang, M. Xu, W. Xie, X. Huang, Y. Chen, J. Wang, DDX18 coordinates nucleolus phase separation and nuclear organization to control the pluripotency of human embryonic stem cells. *Nat. Commun.* **15**, 10803 (2024).

64. X. Wang, G. Hu, L. Wang, Y. Lu, Y. Liu, S. Yang, J. Liao, Q. Zhao, Q. Huang, W. Wang, W. Guo, H. Li, Y. Fu, Y. Song, Q. Cai, X. Zhang, X. Wang, Y.-Q. Chen, X. Zhang, H. Yao, DEAD-box RNA helicase 10 is required for 18S rRNA maturation by controlling the release of U3 snoRNA from pre-rRNA in embryonic stem cells. *Nat. Commun.* **15**, 10303 (2024).

65. B. C. Valdez, L. Perlaky, D. Henning, Expression, cellular localization, and enzymatic activities of RNA helicase II/Guβ. *Exp. Cell Res.* **276**, 249–263 (2002).

66. A. Vanden Broeck, S. Klinge, Principles of human pre-60S biogenesis. *Science* **381**, eadh3892 (2023).

67. K. Sagou, M. Uema, Y. Kawaguchi, Nucleolin is required for efficient nuclear egress of herpes simplex virus type 1 nucleocapsids. *J. Virol.* **84**, 2110–2121 (2010).

68. S. Besse, F. Puvion-Dutilleul, Intranuclear retention of ribosomal RNAs in response to herpes simplex virus type 1 infection. *J. Cell Sci.* **109** (Pt. 1), 119–129 (1996).

69. G. Ouellet Lavallée, A. Pearson, Upstream binding factor inhibits herpes simplex virus replication. *Virology* **483**, 108–116 (2015).

70. D. Walsh, I. Mohr, Assembly of an active translation initiation factor complex by a viral protein. *Genes Dev.* **20**, 461–472 (2006).

71. S. Belin, K. Kindbeiter, S. Hacot, M. A. Albaret, J.-X. Roca-Martinez, G. Thérizols, O. Grosso, J.-J. Diaz, Uncoupling ribosome biogenesis regulation from RNA polymerase I activity during *herpes simplex* virus type 1 infection. *RNA* **16**, 131–140 (2010).

72. K. Yamaguchi, S. Inoue, O. Ohara, T. Nagase, Pulse-chase experiment for the analysis of protein stability in cultured mammalian cells by covalent fluorescent labeling of fusion proteins. *Methods Mol. Biol.* **577**, 121–131 (2009).

73. M. E. Mertens, D. M. Knipe, Herpes simplex virus 1 manipulates host cell antiviral and proviral DNA damage responses. *MBio* **12**, e03552–20 (2021).

74. F. D. Araujo, T. H. Stracker, C. T. Carson, D. V. Lee, M. D. Weitzman, Adenovirus type 5 E4orf3 protein targets the Mre11 complex to cytoplasmic aggresomes. *J. Virol.* **79**, 11382–11391 (2005).

75. B. Dauber, J. Pelletier, J. R. Smiley, The herpes simplex virus 1 vhs protein enhances translation of viral true late mRNAs and virus production in a cell type-dependent manner. *J. Virol.* **85**, 5363–5373 (2011).

76. E. I. Vink, J. Andrews, C. Duffy, I. Mohr, Preventing translational inhibition from ribosomal protein insufficiency by a herpes simplex virus–encoded ribosome-associated protein. *Proc. Natl. Acad. Sci. U.S.A.* **118**, e2025546118 (2021).

77. A. K. Wong, A. Krishnan, O. G. Troyanskaya, GIANT 2.0: Genome-scale integrated analysis of gene networks in tissues. *Nucleic Acids Res.* **46**, W65–W70 (2018).

78. Z. Xie, A. Bailey, M. V. Kuleshov, D. J. B. Clarke, J. E. Evangelista, S. L. Jenkins, A. Lachmann, M. L. Wojciechowicz, E. Kropiwnicki, K. M. Jagodnik, M. Jeon, A. Ma’ayan, Gene set knowledge discovery with Enrichr. *Curr. Protoc.* **1**, e90 (2021).

79. K. K. Lum, T. J. Reed, J. Yang, I. M. Cristea, Differential contributions of interferon classes to host inflammatory responses and restricting virus progeny production. *J. Proteome Res.* **23**, 3249–3268 (2024).

80. J. Y. Kishi, S. W. Lapan, B. J. Beliveau, E. R. West, A. Zhu, H. M. Sasaki, S. K. Saka, Y. Wang, C. L. Cepko, P. Yin, SABER amplifies FISH: Enhanced multiplexed imaging of RNA and DNA in cells and tissues. *Nat. Methods.* **16**, 533–544 (2019).

81. P. J. Thul, L. Åkesson, M. Wiking, D. Mahdessian, A. Geladaki, H. Ait Blal, T. Alm, A. Asplund, L. Björk, L. M. Breckels, A. Bäckström, F. Danielsson, L. Fagerberg, J. Fall, L. Gatto, C. Gnann, S. Hober, M. Hjelmare, F. Johansson, S. Lee, C. Lindskog, J. Mulder, C. M. Mulvey, P. Nilsson, P. Oksvold, J. Rockberg, R. Schutten, J. M. Schwenk, Å. Sivertsson, E. Sjöstedt, M. Skogs, C. Stadler, D. P. Sullivan, H. Tegel, C. Winsnes, C. Zhang, M. Zwahlen, A. Mardinoglu, F. Pontén, K. von Feilitzen, K. S. Lilley, M. Uhlén, E. Lundberg, A subcellular map of the human proteome. *Science* **356**, eaal3321 (2017).

82. M. Uhlén, L. Fagerberg, B. M. Hallström, C. Lindskog, P. Oksvold, A. Mardinoglu, Å. Sivertsson, C. Kampf, E. Sjöstedt, A. Asplund, I. Olsson, K. Edlund, E. Lundberg, S. Navani, C. A.-K. Szigyarto, J. Odeberg, D. Djureinovic, J. O. Takanen, S. Hober, T. Alm, P.-H. Edqvist, H. Berling, H. Tegel, J. Mulder, J. Rockberg, P. Nilsson, J. M. Schwenk, M. Hamsten, K. von Feilitzen, M. Forsberg, L. Persson, F. Johansson, M. Zwahlen, G. von Heijne, J. Nielsen, F. Pontén, Tissue-based map of the human proteome. *Science* **347**, 1260419 (2015).

83. N. H. Cho, K. C. Cheveralls, A.-D. Brunner, K. Kim, A. C. Michaelis, P. Raghavan, H. Kobayashi, L. Savy, J. Y. Li, H. Canaj, J. Y. S. Kim, E. M. Stewart, C. Gnann, F. McCarthy, J. P. Cabrera, R. M. Brunetti, B. B. Chhun, G. Dingle, M. Y. Hein, B. Huang, S. B. Mehta, J. S. Weissman, R. Gómez-Sjöberg, D. N. Itzhak, L. A. Royer, M. Mann, M. D. Leonetti, OpenCell: Endogenous tagging for the cartography of human cellular organization. *Science* **375**, eabi6983 (2022).

84. S. A. Quinodoz, L. Jiang, A. A. Abu-Alfa, T. J. Comi, H. Zhao, Q. Yu, L. W. Wiesner, J. F. Botello, A. Donlic, E. Soehalim, C. Zorbas, L. Wacheul, A. Košmrlj, D. L. J. Lafontaine, S.

Klinge, C. P. Brangwynne, Mapping and engineering RNA-controlled architecture of the multiphase nucleolus. bioRxiv [Preprint] (2024). https://doi.org/10.1101/2024.09.28.615444.
